# Geographic and demographic patterns of cervical cancer in Africa using GLOBOCAN 2022

**DOI:** 10.1186/s12885-026-16000-7

**Published:** 2026-04-18

**Authors:** Xiaoli Zhou, Hong Xu, Yu Fu, Qiqin Nie, Baohua Zhang, Xiaoya Guo, Mingzhu Hu

**Affiliations:** 1 Department of Obstetrics and Gynecology, Punan Branch of Renji Hospital, Shanghai Jiaotong University School of Medicine (Punan Hospital in Pudong new district, Shanghai), Shanghai, China; 2 Department of General Practice, Pujiang Community Health Service Center, Minhang District, Shanghai, China

**Keywords:** Human Development Index, Health disparities, Burden projection, Epidemiology, Cancer control

## Abstract

**Background:**

Cervical cancer remains a significant public health challenge for women in Africa. This study aimed to quantify the current burden of cervical cancer across the continent using the most recent estimates from the Global Cancer Observatory (GLOBOCAN) 2022.

**Methods:**

Data were obtained from GLOBOCAN 2022. Age-standardized incidence rates (ASIR) and age-standardized mortality rates (ASMR) per 100,000 person-years were calculated using direct standardization with the Segi–Doll world standard population. Pearson’s correlation analysis was applied to examine associations between the Human Development Index (HDI) and cervical cancer incidence and mortality. Future numbers of cases and deaths through 2050 were projected based on global population forecasts.

**Results:**

In 2022, an estimated 125,699 new cervical cancer cases and 80,614 deaths occurred in Africa. Nigeria recorded the highest numbers of incident cases (13,676) and deaths (7,093). The highest ASIR was observed in Eswatini (95.9 per 100,000), while Malawi exhibited the highest ASMR (54.1 per 100,000). Mortality rates increased progressively with age and rose sharply among individuals aged 75 years and older. A negative association was identified between HDI and mortality rates. Projections indicate that Nigeria and Tanzania will experience the largest future burden, with Equatorial Guinea, Tanzania, and Zambia facing increases exceeding 200% in incidence and 210% in mortality by 2050.

**Conclusions:**

Cervical cancer mortality remains disproportionately high in countries with lower socioeconomic development. Targeted and timely interventions in these settings are essential to curb the projected escalation of disease burden.

**Supplementary Information:**

The online version contains supplementary material available at 10.1186/s12885-026-16000-7.

## Introduction

In 2022, cervical cancer ranked as the fourth most commonly diagnosed cancer and the fourth leading cause of cancer death among women worldwide, following breast, lung, and colorectal cancers [1], with an estimated 662,044 new cases (age-standardized incidence rate [ASIR]: 14.12/100,000) and 348,709 deaths (age-standardized mortality rate [ASMR]: 7.08/100,000) [2]. Persistent infection with oncogenic human papillomavirus (HPV), particularly types 16 and 18, which account for 71% of cases, is the primary etiological factor [3]. Cervical cancer follows a well-defined progression from HPV infection to precancerous lesions and invasive disease, with additional cofactors—such as tobacco use, HIV infection, early sexual debut, long-term oral contraceptive use, and multiple sexual partners—further increasing risk [4–7]. Together, these features indicate that cervical cancer is largely preventable and, when detected early, highly treatable, underscoring the critical role of effective screening programs in reducing disease burden and improving outcomes [8].

Africa carries a disproportionate burden of cervical cancer, accounting for a large share of global incidence and mortality [9]. Although rates have declined markedly in high-income countries following widespread screening, HPV vaccination, and improved treatment, nearly 90% of cases and deaths now occur in low- and middle-income countries, with 19 of the 20 highest-incidence countries located in Africa [10]. Persistently low HPV vaccination coverage, limited screening capacity, and restricted access to timely treatment remain major contributors to this excess burden, particularly in sub-Saharan Africa [10]. Moreover, the high prevalence of HIV in several regions further increases cervical cancer risk and accelerates disease progression, leading to poorer outcomes [11].

A clearer understanding of the contemporary epidemiology of cervical cancer in Africa is essential for monitoring progress toward the World Health Organization’s cervical cancer elimination targets—including the 90‑70‑90 targets for vaccination, screening, and treatment by 2030—and for informing equitable allocation of prevention and treatment resources [12]. Although temporal and geographic variations in cervical cancer burden have been reported [11–15], current and detailed assessments of the burden in African countries—particularly in the post–COVID-19 period—remain limited. This data gap constrains the development and implementation of targeted and effective public health interventions. Therefore, this study provides a dedicated Africa-focused assessment, integrates HDI-stratified and age-specific analyses, and includes country-level forward projections to 2050, providing evidence to support targeted and context‑specific cervical cancer control strategies on the continent.

## Materials and methods

### Data sources

Data on incident cases and deaths from cervical cancer (C53), as defined by the International Classification of Diseases, 10th Revision (ICD-10), were obtained from the Global Cancer Observatory (GLOBOCAN) 2022 database, covering 185 countries and territories. (https://gco.iarc.fr/) [1]. GLOBOCAN, maintained by the International Agency for Research on Cancer (IARC), provides standardized estimates of cancer incidence, mortality, and prevalence for 36 cancer types worldwide [16, 17]. At the time of analysis, GLOBOCAN 2022 represented the most recent globally comparable dataset released by IARC, and no newer worldwide estimates had been published. Population data for 2022 were sourced from the World Bank (https://data.worldbank.org/). Country-level socioeconomic development was assessed using the Human Development Index (HDI), a composite measure combining life expectancy, education, and per capita income. Countries were categorized into four tiers: very high (≥ 0.800), high (0.700–0.799), medium (0.550–0.699), and low (< 0.550) HDI [12].

### Variable interpretation

Key variables included incident cases, deaths, ASIR, ASMR, and age/HDI stratifications. Incidence was defined as the number of newly diagnosed cervical cancer cases within a specified population and time period, expressed as counts or rates per 100,000 person-years. These rates reflect the average risk of developing cervical cancer in 2022 and enable comparisons across countries and regions. Incidence data were derived primarily from population-based cancer registries. Mortality was defined as the number of deaths attributable to cervical cancer within a given time frame, with mortality rates expressed per 100,000 person-years, based on data from national statistical systems.

### Burden projections

Projections of cervical cancer incidence and mortality from 2022 to 2050 were derived using the IARC CANCER TOMORROW tool (https://gco.iarc.fr/tomorrow/en), based on GLOBOCAN 2022 estimates for 36 cancer types across 185 countries and territories [1]. The tool projects future cases and deaths by applying 2022 age-specific incidence and mortality rates to United Nations (UN) World Population Prospects data. The primary projection scenario assumes constant national age-specific rates (0% annual change), such that future burden is driven solely by population growth and ageing. Additional alternative scenarios assuming annual rate changes of ± 1%, ± 2%, and ± 3% were examined as sensitivity analyses to illustrate potential departures from the constant-rate assumption. These projections assume that 2022 rates reflect current risk patterns and that UN population forecasts accurately represent demographic trends through 2050 [18]. The constant-rate scenario represents a demographic projection rather than an intervention-based forecast and was used as the main reference scenario in this study.

### Statistical analysis

ASIR and ASMR were calculated using direct standardization with the Segi–Doll world standard population as the reference [19, 20]. Pearson correlation was used as the primary method to assess associations between ASIR, ASMR, and HDI. Prior to applying Pearson correlation, the distributions of the variables and the assumption of approximate linearity were examined. Sensitivity analyses using Spearman rank correlation were also conducted to confirm the robustness of the results. All analyses were performed using R software (version 4.3.3), including the packages dplyr (version 1.1.4), rnaturalearth (version 0.3.4), countrycode (version 1.5.0), tidyverse (version 2.0.0), ggplot2 (version 3.4.4), and Hmisc (version 5.1-1.1.1), with a two-sided P value < 0.05 considered statistically significant.

## Results

### Geographic variations in cervical cancer incidence and mortality across Africa in 2022

**Fig. 1:**
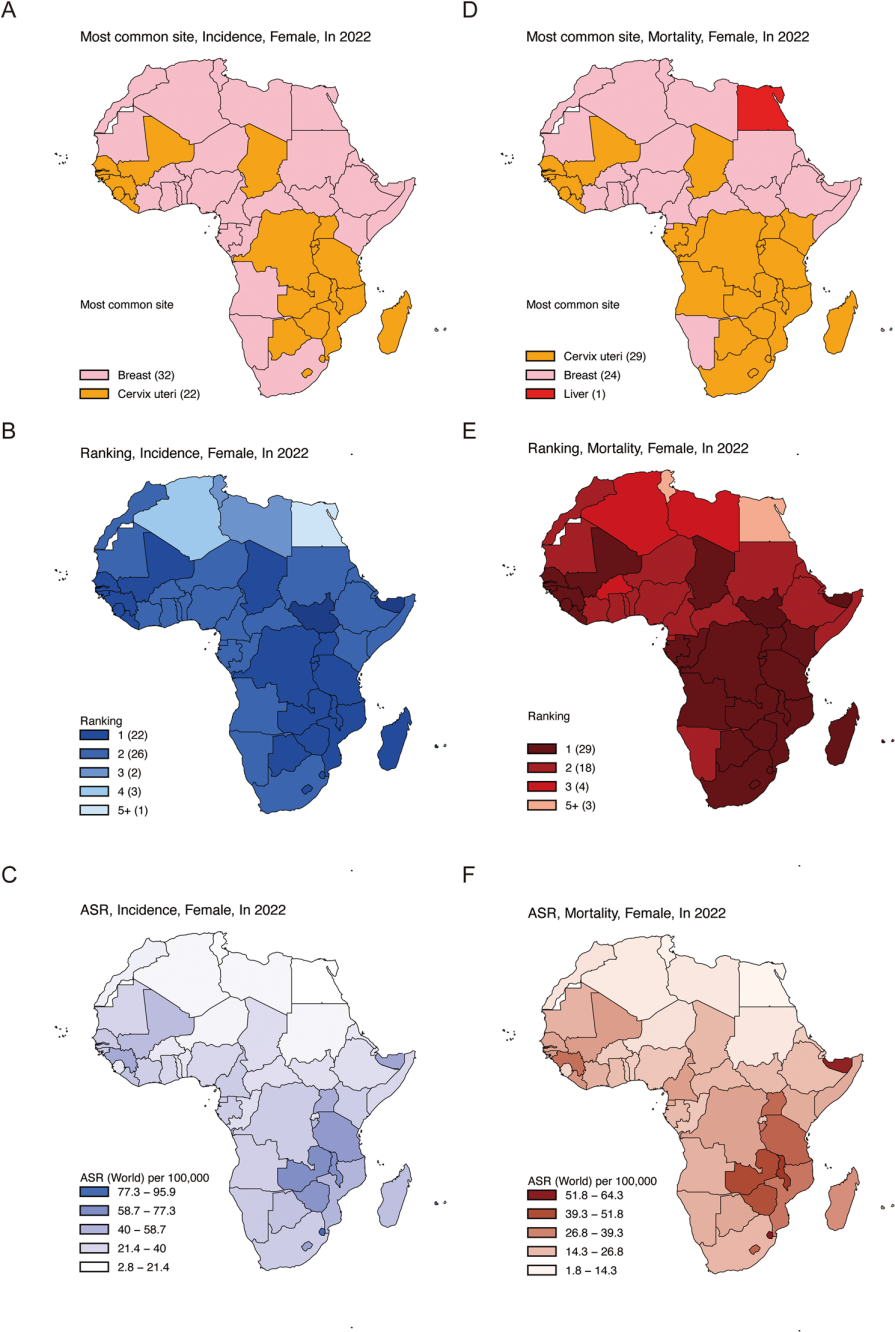
African region of absolute number of cervical cancer cases. Most common site per country of incidence (**A**), cervical cancer ranking (**B**) and ASR (World) per 100,000 of incidence **C**. Most common site per country of mortality (**D**), cervical cancer ranking (**E**) and ASR per 100,000 of mortality **F**. The data was obtained from the GLOBOCAN database in 2022. *ASR* Age-standardized rate

In 2022, an estimated 125,699 new cervical cancer cases occurred in Africa, accounting for 19.0% of the global total (Table 1). Cervical cancer was the most frequently diagnosed cancer among women in 22 of 54 African countries (Fig. 1A) and ranked second in 26 countries (Fig. 1B). The continent-wide ASIR was 26.4 per 100,000 population (Table 1; Fig. 1C). During the same year, Africa recorded approximately 80,614 cervical cancer deaths, representing 23.1% of global mortality from the disease (Table 1). Cervical cancer was the leading cause of cancer-related death among women in 29 countries (Fig. 1D) and ranked among the top three causes in 51 countries (Fig. 1E). The overall ASMR was 17.6 per 100,000 population (Table 1; Fig. 1F).

**Table 1:** New cases and deaths, ASIR and ASMR rates (per 100,000) of cervical cancer in Africa, 2022. The data sources were obtained from the GLOBOCAN cancer today database in 2022. *ASIR* Age-standardized incidence rate, *ASMR* Age-standardized mortality rate, *HDI* Human Development Index

Location	ASIR	New cases	ASMR	Deaths	Region
World	14.10	662,301	7.10	348,874	
Africa	26.40	125,699	17.60	80,614	
Morocco	11.97	2644	6.62	1468	North Africa
Algeria	7.96	1799	4.58	1013	North Africa
Egypt	2.77	1302	1.77	820	North Africa
Sudan	8.60	1234	5.20	738	North Africa
Tunisia	5.25	414	2.62	210	North Africa
Libya	8.00	278	5.14	169	North Africa
South Africa	33.18	10,532	19.03	5976	Southern Africa
Malawi	70.85	4701	54.07	3340	Southern Africa
Zimbabwe	68.20	3520	47.93	2318	Southern Africa
Lesotho	60.49	598	42.27	413	Southern Africa
Botswana	39.06	454	64.33	269	Southern Africa
Eswatini	95.89	417	22.77	253	Southern Africa
Namibia	33.45	350	20.48	203	Southern Africa
Tanzania	64.75	10,868	42.19	6832	East Africa
Ethiopia	22.28	8168	16.82	5975	East Africa
Uganda	53.76	6938	40.58	4782	East Africa
Kenya	32.83	5845	36.91	4000	East Africa
Mozambique	47.79	5456	21.38	3591	East Africa
Madagascar	41.77	4060	30.02	2690	East Africa
Zambia	71.50	3640	49.39	2285	East Africa
Burundi	43.58	1457	35.04	1081	East Africa
Somalia	26.58	1167	21.78	919	East Africa
Rwanda	18.92	866	13.79	609	East Africa
South Sudan	21.42	749	17.55	593	East Africa
Eritrea	16.41	196	12.73	150	East Africa
Comoros	52.01	163	35.68	102	East Africa
Mauritius	12.90	136	5.70	67	East Africa
France, La Ré	10.97	75	12.98	54	East Africa
Djibouti	16.53	71	5.09	39	East Africa
Nigeria	26.18	13,676	14.25	7093	West Africa
Ghana	27.00	3072	25.69	1837	West Africa
Angola	30.40	2823	16.85	1815	West Africa
Guinea	55.01	2551	20.22	1715	West Africa
Cameroon	33.11	2525	38.88	1695	West Africa
Mali	43.14	2436	20.38	1461	West Africa
Côte d’Ivoire	32.04	2360	26.09	1431	West Africa
Senegal	34.26	2064	23.70	1327	West Africa
Burkina Faso	15.88	988	12.96	775	West Africa
Liberia	39.61	717	28.28	478	West Africa
Benin	18.12	701	12.75	475	West Africa
Niger	9.31	624	7.06	440	West Africa
Togo	19.05	511	13.30	334	West Africa
Sierra Leone	16.46	486	19.49	302	West Africa
Mauritania	28.29	468	10.85	292	West Africa
Gambia	39.37	325	27.41	204	West Africa
Guinea-Bissau	34.30	224	25.87	157	West Africa
Cape Verde	16.07	46	9.78	27	West Africa
DR Congo	32.91	8705	24.56	6187	Central Africa
Chad	23.51	1111	18.38	841	Central Africa
Congo, Republic of	22.32	397	14.20	248	Central Africa
Central Africa Rep	21.77	295	18.31	240	Central Africa
Gabon	32.48	271	17.77	139	Central Africa
Equatorial Guinea	33.23	127	21.86	76	Central Africa
Sao Tome	18.77	14	13.74	10	Central Africa
Low HDI	31.17	77,833	21.44	51,126	
Medium HDI	34.00	32,792	22.81	20,885	
High HDI	13.06	14,915	7.55	8508	
Very high HDI	11.00	75	5.10	39	

At the national level, Nigeria reported the highest numbers of both incident cases (13,676) and deaths (7,093), with an ASIR of 26.2 and an ASMR of 14.3 per 100,000. Eswatini exhibited the highest ASIR (95.9 per 100,000), followed by Zambia (71.5 per 100,000) and Malawi (70.9 per 100,000). Mortality rates were particularly elevated in Botswana and Malawi, with ASMRs of 64.3 and 54.1 per 100,000, respectively. In contrast, Egypt had the lowest ASIR (2.8 per 100,000), followed by Tunisia (5.3 per 100,000) and Algeria (8.0 per 100,000), which also reported the lowest ASMRs (1.8, 2.6, and 4.6 per 100,000, respectively; Table 1; Fig. 1C and F, and Figure S1).

### Cervical cancer incidence and mortality stratified by age

**Fig. 2:**
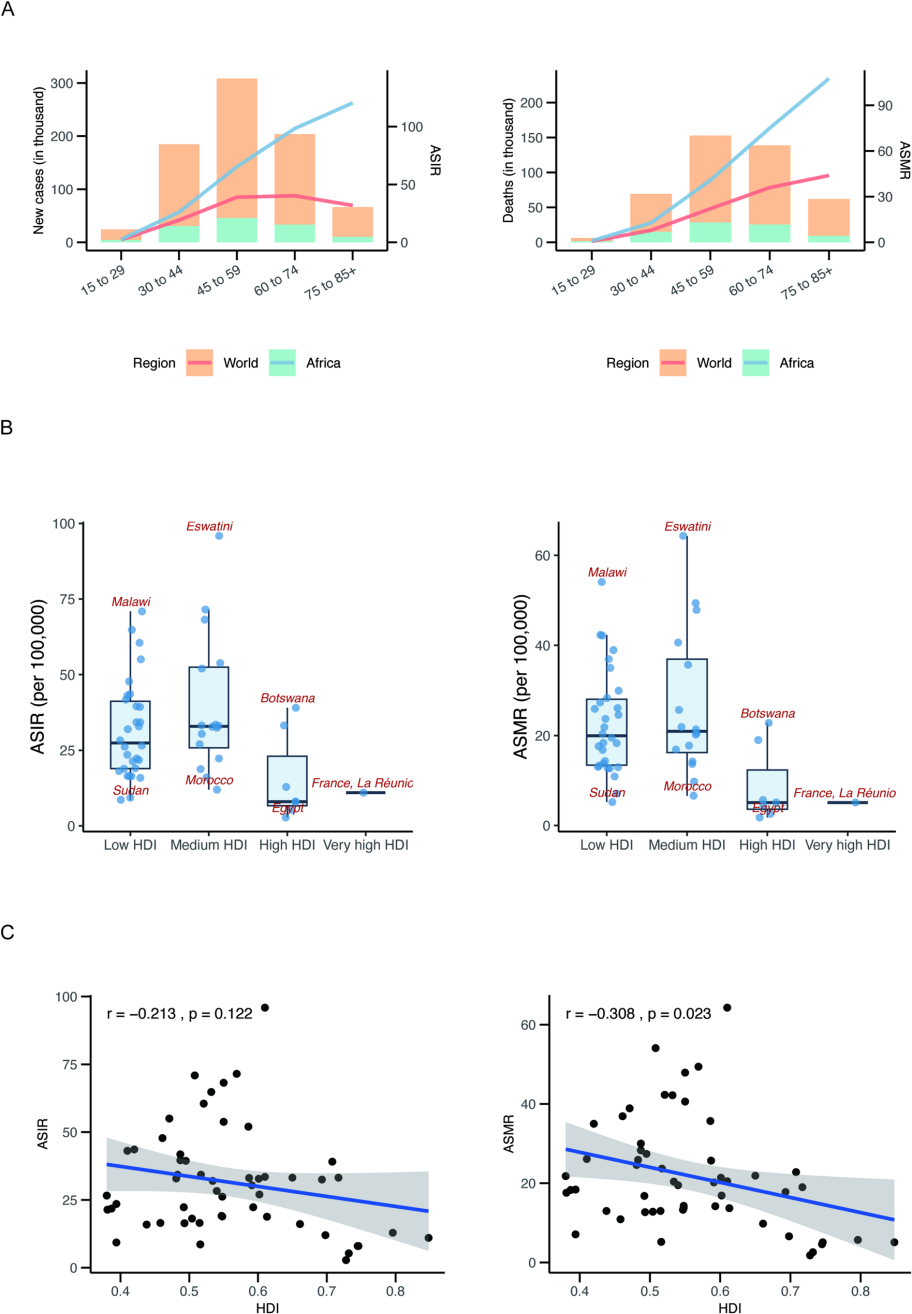
Incidence and mortality of cervical cancer in Africa across different age groups and Human Development Index. **A** The lines represent the number of new cases, the number of deaths, ASIR and ASMR (per 100,000) trends of cervical cancer for world and Africa across age groups. **B** ASIR and ASMR (per 100,000) of cervical cancer by HDI in African countries. **C** The correlation analysis of ASIR and ASMR (per 100,000) with HDI. The data was obtained from the GLOBOCAN database in 2022. *ASIR* Age-standardized incidence rate, *ASMR* Age-standardized mortality rate

In Africa, an estimated 45,938 new cervical cancer cases and 28,672 deaths occurred among women aged 45–59 years. The ASMR increased markedly from ages 30–44, indicating a substantial disease impact in younger adult women. Compared with global averages, Africa exhibited higher ASIR for cervical cancer across individuals aged 15 years and older, accompanied by consistently higher mortality rates in the same age groups (Fig. 2A; Table S1). Notably, the age group with the highest incidence varied across countries (Figure S2). Seven countries—Libya, Eswatini, Malawi, South Africa, Mozambique, Sierra Leone, and Chad—showed the highest ASIR in the 45–59-year age group (Table S2). Among these, Malawi, Mozambique, Sierra Leone, and Chad also experienced the highest in ASMR, occurring in women aged 60–74 years. Overall, cervical cancer mortality increased progressively with age, reaching its highest levels among individuals aged 75 years and older in most countries (Figure S2). The highest ASMRs in this age group were observed in Tanzania (456.1 per 100,000), Zambia (399.1 per 100,000), Zimbabwe (290.2 per 100,000), and Eswatini (269.6 per 100,000; Table S2).

### Cervical cancer incidence and mortality stratified by human development index

Nearly 89% of cervical cancer cases in Africa were diagnosed in countries with low or medium HDI (Table 1). Figure 2B and C illustrate cervical cancer incidence and mortality rates stratified by HDI at both regional and national levels, as well as the associations between HDI and age-standardized rates. Countries classified as having very high or high HDI exhibited lower ASIRs, at 11.0 and 13.1 per 100,000, respectively, whereas substantially higher incidence rates were observed in low- and medium-HDI countries (31.2 and 34.0 per 100,000, respectively). Similarly, ASMRs were highest in low-HDI (21.4 per 100,000) and medium-HDI (22.8 per 100,000) settings (Fig. 2B; Table 1). Overall, a moderate inverse association was identified between HDI and ASMR (Pearson’s *r* = − 0.308, *P* = 0.023), with the strongest correlation observed among individuals aged 45–59 years (Fig. 2C; Figure S3). In contrast, no statistically significant association was detected between HDI and ASIR at the continental level (Fig. 2C).

### Projected burden of cervical cancer in Africa by 2050

**Fig. 3:**
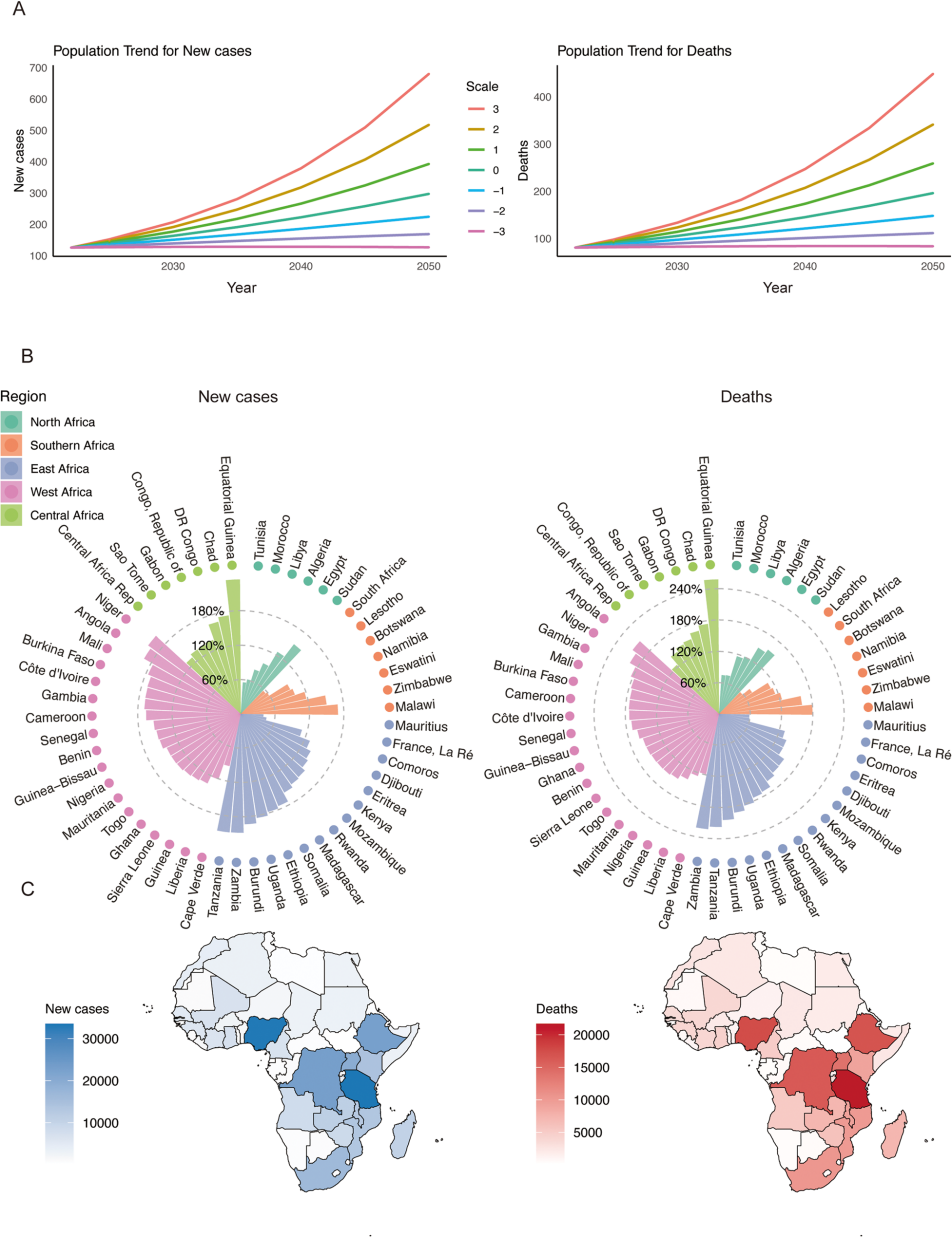
Forecast of cervical cancer cases in Africa. **A** Projected new cases and deaths under the primary constant-rate scenario and alternative ± 1–3% annual rate change scenarios. Lines beginning at 2022 indicate the observed baseline, while values from 2025 to 2050 represent forecasts of new cases and deaths based on GLOBOCAN 2022 data. The scale reflects the annual percentage change in the number of cases or deaths relative to the 2022 baseline. **B** The circular bar plots depict the projected percentage change in cervical cancer absolute new case and death counts from 2022 to 2050, assuming age-specific rates remain constant at 2022 baseline levels. Bars are color-coded according to region. **C** The maps illustrate the projected absolute changes in cervical cancer incidence and mortality between 2022 and 2050, assuming age-specific rates remain constant at 2022 baseline levels

Assuming age-specific incidence and mortality rates remain at 2022 levels, Africa is projected to record approximately 297,250 new cervical cancer cases and 196.050 deaths by 2050 (Table S3; Fig. 3A). To offset population-driven increases and maintain stable absolute numbers, an average annual reduction of approximately 3% in both incidence and mortality rates would be required (Table S3).

By 2050, 43 African countries (79.6%) are expected to experience substantial increases in cervical cancer burden, with projected rises of 100% or more in case numbers (Fig. 4B; Table S4). The largest absolute burdens are anticipated in Nigeria, Tanzania, South Africa, Ethiopia, Uganda, Mozambique, and the Democratic Republic of the Congo (Fig. 3C). Tanzania is projected to report the highest numbers of incident cases (33,488) and deaths (21,677), reflecting its large population size, followed by Nigeria with an estimated 33,138 new cases and 17,566 deaths. Marked relative increases are projected in Equatorial Guinea (234.7%), Tanzania (208.1%), and Zambia (207.3%). Corresponding mortality is also expected to rise sharply in these countries, with increases exceeding 210%. In contrast, more moderate growth is projected for countries such as France, La Réunion, Mauritius, and Lesotho (Figure S4; Table S4).

## Discussion

In this comprehensive evaluation of cervical cancer burden in Africa, we observed marked geographic disparities in incidence and mortality in 2022, with southern and eastern African countries experiencing the highest rates. Women aged 45–59 years bore a disproportionate share of disease burden, while mortality escalated sharply in older age groups. A large majority of cases occurred in low and medium HDI settings, and a moderate inverse association was seen between HDI and mortality. Projections indicate that without substantial improvements in prevention and care, cervical cancer incidence and mortality could more than double in most African countries by 2050.

This study revealed substantial geographic heterogeneity in cervical cancer incidence and mortality across Africa, with the highest burdens concentrated in countries such as Nigeria, Eswatini, Malawi, and Tanzania. Recent population‑based cancer registry analyses strengthen evidence of regional disparities in cervical cancer in Africa. A study of cancer registry data from Nigeria found persistent high incidence in multiple regions, reflecting limited screening and diagnostic capacities across states [21]. Hospital-based data from western Kenya show that cervical cancer patients present at advanced stages and exhibit high mortality, reflecting regional disparities in cancer outcomes [22]. These studies collectively confirm that regional clusters of high cervical cancer burden are not artifacts of global estimates but reflect real, empirically observed patterns.

Our age-stratified analysis aligns with clinical studies showing that African women frequently present with advanced-stage cervical cancer, particularly in older age groups. In Ethiopia, over 70% of women were diagnosed at stage II or higher, with older age strongly associated with later-stage disease [23]. Similarly, Tanzanian women aged ≥ 50 were more likely to present at late stages [24], and sub-Saharan Africa multi-site audits showed that age ≥ 65 years was linked with lymph node involvement and reduced survival [25]. These observations help explain the steeply rising mortality observed among older age groups in our population-level data.

High prevalence of oncogenic HPV, particularly among women living with HIV, is an important biological and epidemiological factor contributing to cervical cancer in Africa [26, 27]. Although our analysis did not include HIV prevalence or treatment data—since GLOBOCAN 2022 and CANCER TOMORROW estimates do not incorporate HIV-specific covariates—the observed high incidence and mortality in several African countries is consistent with epidemiological evidence showing that HIV-positive women have a higher prevalence of high-risk HPV infection and an elevated risk of progression to high-grade cervical lesions [28–30]. By referencing these studies, we provide context for the disparities observed across countries without implying direct modeling of HIV effects in our projections.

Low cervical cancer screening coverage contributes substantially to delayed diagnosis and high mortality. DHS analyses in multiple sub-Saharan countries show that lifetime screening coverage among women aged 30–49 ranged only 4–28% [31]. In Ethiopia, only 16% of eligible women were ever screened, with rural residence and low education as strong predictors of non-uptake [32]. In women living with HIV, screening coverage remained below 30% despite frequent healthcare interactions [31]. These findings explain part of the high mortality and late-stage presentation in our analysis.

Despite evidence that HPV vaccination effectively reduces high-risk HPV infections, coverage in Africa remains suboptimal. A recent review reported national coverage below 50% in many sub-Saharan countries, with supply chain and logistical barriers cited [33]. Rwanda’s school-based program achieved higher coverage, but sustainability remains limited by financing and delivery constraints [34]. Pilot programs in Kenya and South Africa face similar implementation challenges [33, 35, 36]. In addition to logistical constraints, cultural and sociological concerns—including misconceptions that HPV vaccination affects fertility or promotes reduced birth rates—have been reported in several settings and may reduce vaccine acceptance [37, 38]. Expanding HPV vaccination to boys offers an additional opportunity to reduce HPV transmission and future cervical cancer risk among women [39], but program implementation must also consider cultural acceptance and equitable access.

Some medium-HDI countries showed higher ASIR and ASMR than high-HDI countries. The stronger inverse association between HDI and mortality compared with incidence likely reflects a combination of limited but partial screening and diagnostic coverage, higher prevalence of risk factors such as HPV and HIV infection, and population age structure, rather than higher underlying biological risk. In contrast, high-HDI countries benefit from widespread vaccination, organized screening, and timely treatment, which reduce both incidence and mortality. Notably, the negative correlation between mortality and HDI is clearer than that for incidence because mortality depends heavily on timely diagnosis and access to effective treatment, whereas incidence is influenced both by underlying risk and by the extent of detection. In Nigeria, poorer regions with limited diagnostic access experienced higher case-fatality rates than wealthier regions [40, 41]. In Ethiopia, restricted access to radiotherapy and pathology services in rural areas resulted in lower survival compared with urban populations [42, 43]. These studies indicate that socioeconomic and health system disparities amplify mortality independently of incidence.

Demographic projections indicate that, without improvements in prevention, screening, and treatment, cervical cancer cases and deaths in Africa will rise sharply by 2050. Population growth and aging alone could double the current burden, consistent with modeling studies that integrate demographic change and risk factor profiles [2, 44]. Our findings underscore the urgent need for targeted interventions, including expanded screening, vaccination, and strengthened health system capacity, to mitigate this anticipated increase.

This study has several limitations. Although the analyses relied on high-quality data from population-based cancer registries, caution is needed when interpreting estimates for countries with sparse registry coverage, where national figures were often inferred from proxy sources [1]. In many low- and middle-HDI African countries, incomplete surveillance systems may compromise the accuracy of reported cancer incidence and mortality, potentially overlooking local heterogeneity. The persistent gaps and errors in existing registries indicate that the true cancer burden in these regions is likely underestimated. Employing mixed-methods approaches—including community-based surveys, policy analyses, case studies, and focus group discussions—could provide more detailed and actionable insights into local contexts [45]. Moreover, disruptions caused by the COVID-19 pandemic have reduced screening activities and delayed cervical cancer detection [46–48], creating a cohort of women at heightened risk. Leveraging healthcare and surveillance infrastructures established for COVID-19 could strengthen national cervical cancer monitoring and improve the delivery of preventive services.

Finally, the GLOBOCAN 2022 estimates are derived from historical trends and may not fully capture the current cancer landscape; subsequent comparisons with contemporaneous national data will be essential to evaluate potential discrepancies and quantify pandemic-related impacts. Projections assume that 2022 ASIR and ASMR remain constant and do not incorporate potential changes in vaccination, screening, or treatment coverage. Therefore, they should be interpreted as demographic scenario projections reflecting the population-driven burden under a status-quo rate assumption, rather than as forecasts accounting for future interventions. Overall, the study underscores persistent health disparities and identifies high-risk populations and regions that should be prioritized in public health planning. Timely, context-specific interventions are essential to mitigate the projected rise in cervical cancer burden and reduce avoidable deaths among African women.

## Supplementary Information

Supplementary Material 1.

## Data Availability

The data that support the findings of this study are available in GLOBOCAN 2022 database (https://gco.iarc.fr/), World Bank website (https://data.worldbank.org/) and the United Nations Development Program (UNDP) database (https://hdr.undp.org/). Further information is available from the corresponding author upon request.

## Abbreviations

HDI: Human development index
ASR: Age-standardized rates
ASIR: Age-standardized incidence rates
ASMR: Age-standardized mortality rates
