# Supplementary material for: Geographic and demographic patterns of cervical cancer in Africa using GLOBOCAN 2022
Source: BMC Cancer. 2026 Apr 18;26:689. doi: 10.1186/s12885-026-16000-7 (PMC13220435; doi:10.1186/s12885-026-16000-7)
Supplement: Supplementary file 1 — Supplementary Material 1. [file 12885_2026_16000_MOESM1_ESM.docx]

**Assessing the African Burden of Cervical Cancer: A Demographic Analysis Using GLOBOCAN 2022**

**Running title: Cervical cancer and Africa**


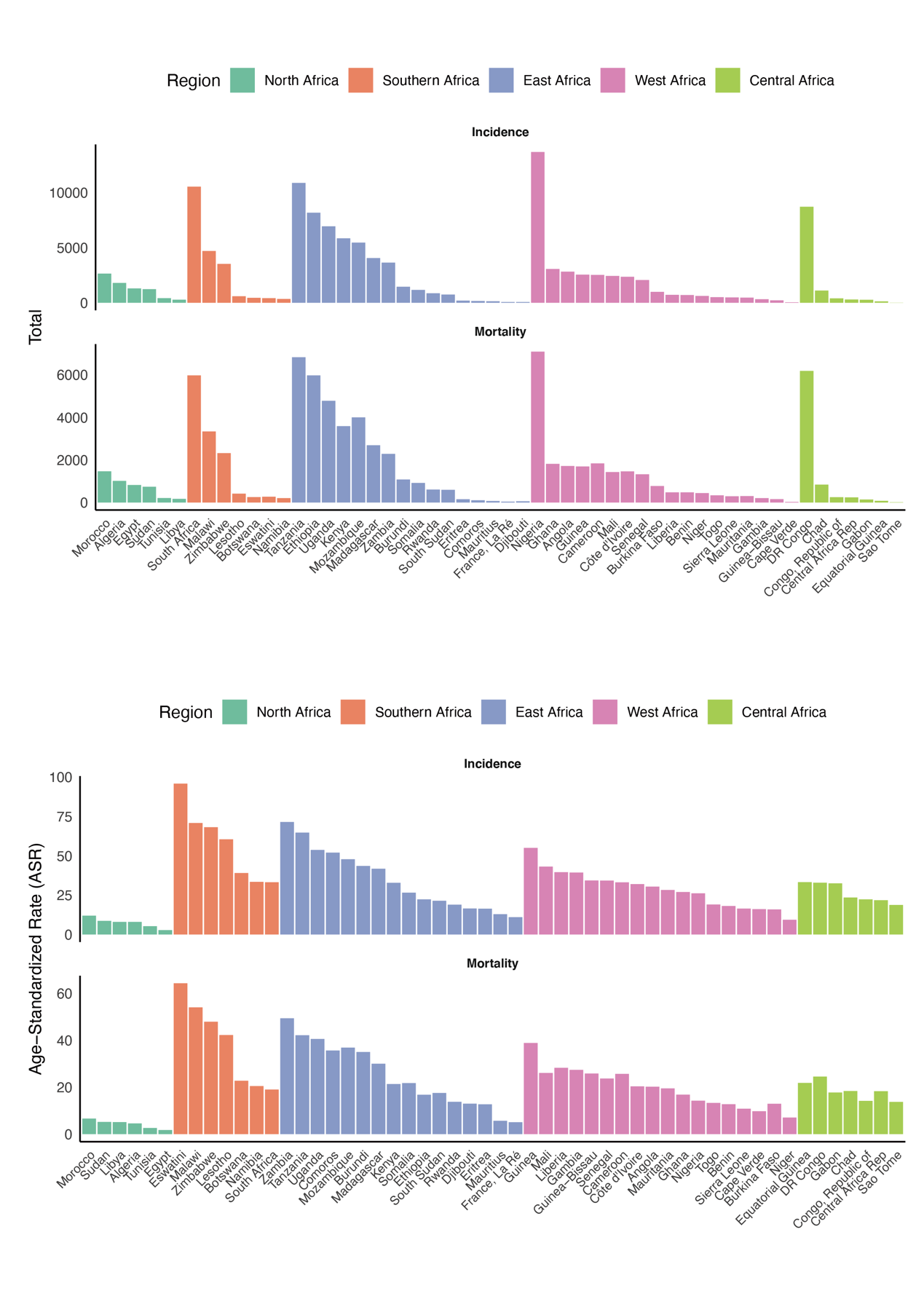


**Figure S1.** **Cervical cancer incidence and mortality in African countries in 2022.** Bars are colored by region. The data was obtained from the GLOBOCAN database in 2022. ASR, age-standardized rate.


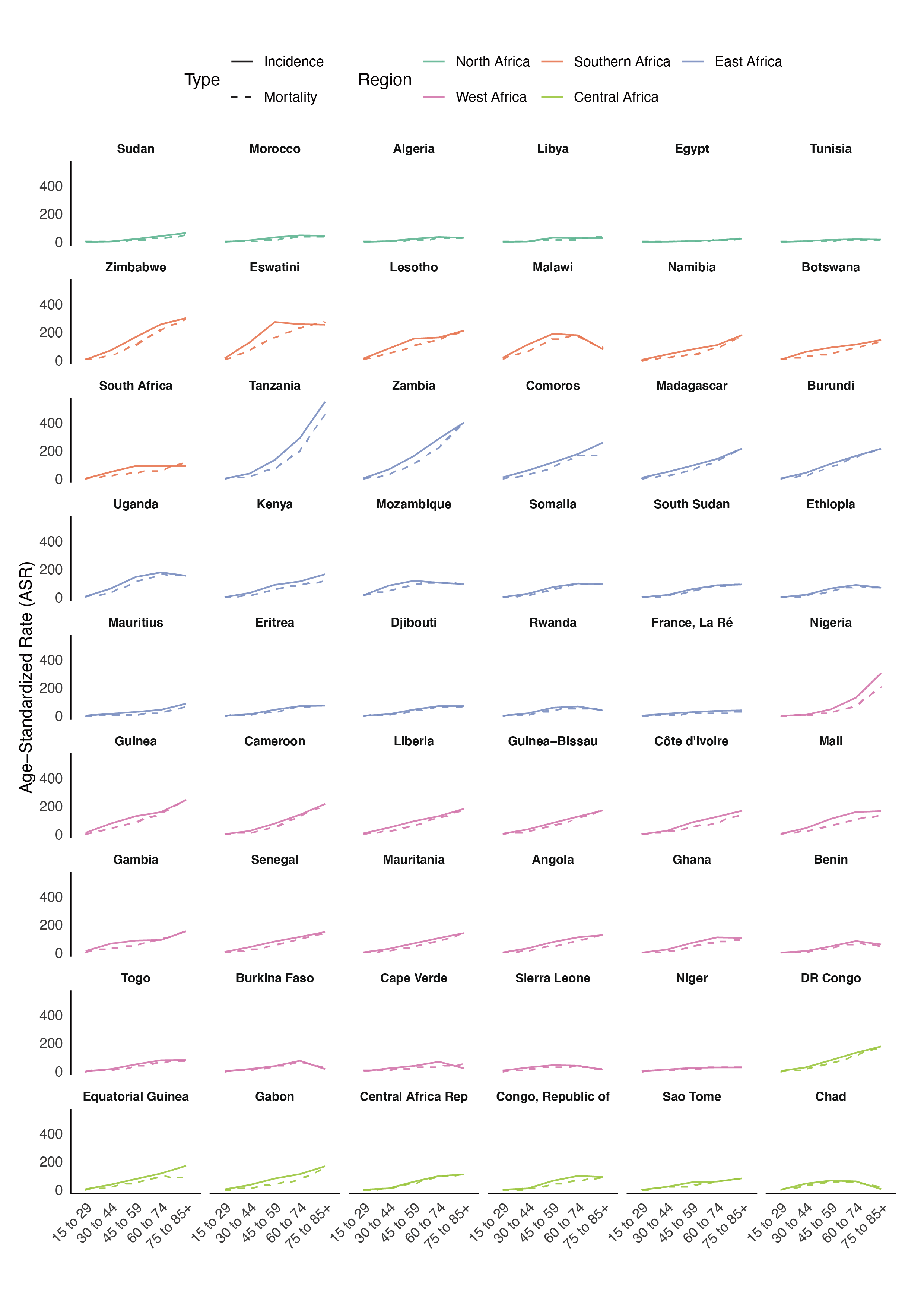


**Figure S2. Age-specific distribution of cervical cancer incidence and mortality in African countries in 2022.** Lines are colored by region. The data was obtained from the GLOBOCAN database in 2022.

**
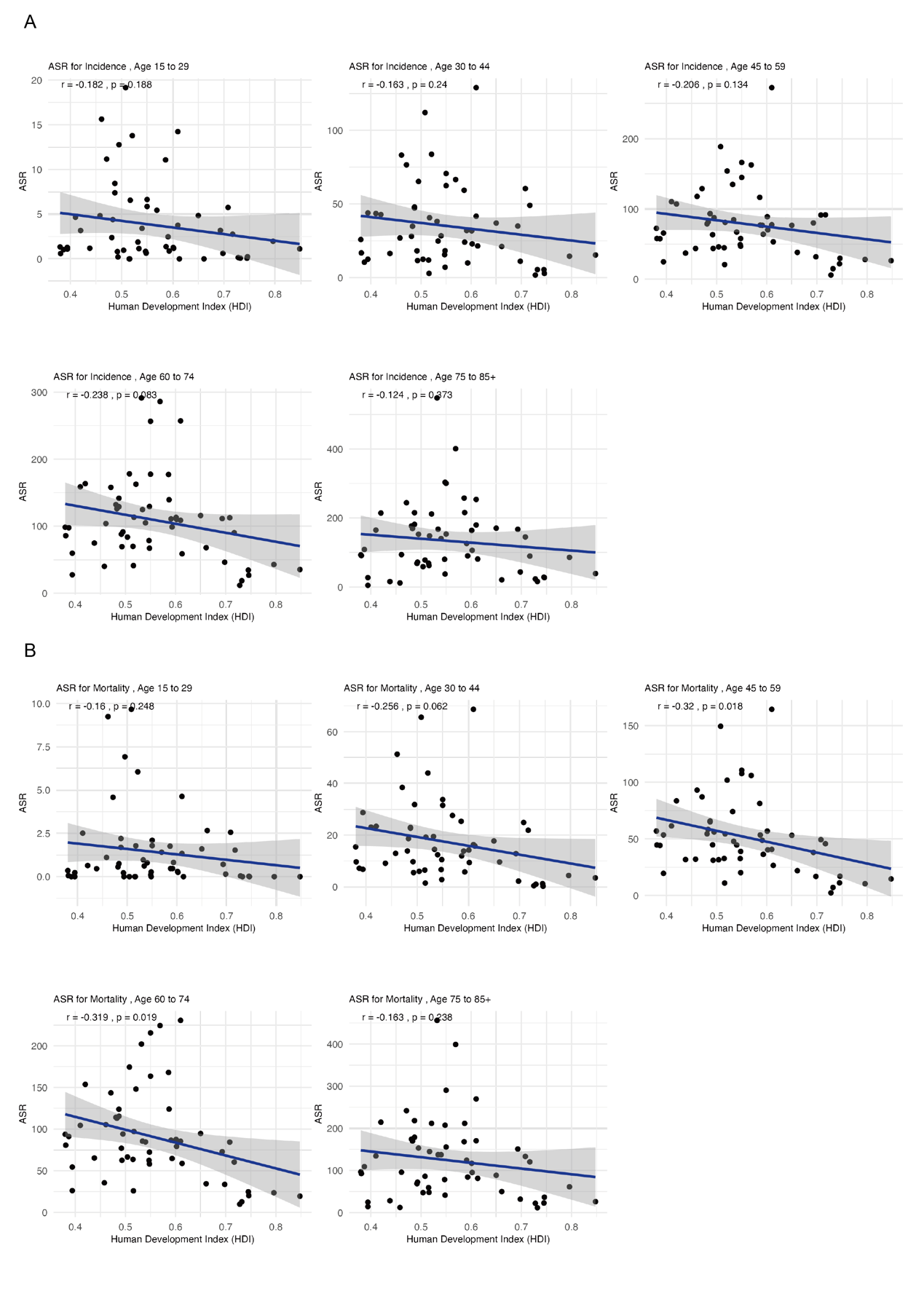
**

**Figure S3. Scatter plot showing the correlation between the Human Development Index and cervical cancer incidence or mortality rates across different age groups.** The solid lines indicate the linear regression fit, reflecting the trend between HDI and ASR for each group. Shaded areas around the lines represent the 95% confidence intervals, illustrating the uncertainty of the trend. Each point corresponds to a country, with colors or symbols distinguishing between age groups and whether the ASR pertains to incidence or mortality. Data was sourced from the GLOBOCAN database in 2022. HDI, Human Development Index; ASR, age-standardized rate.


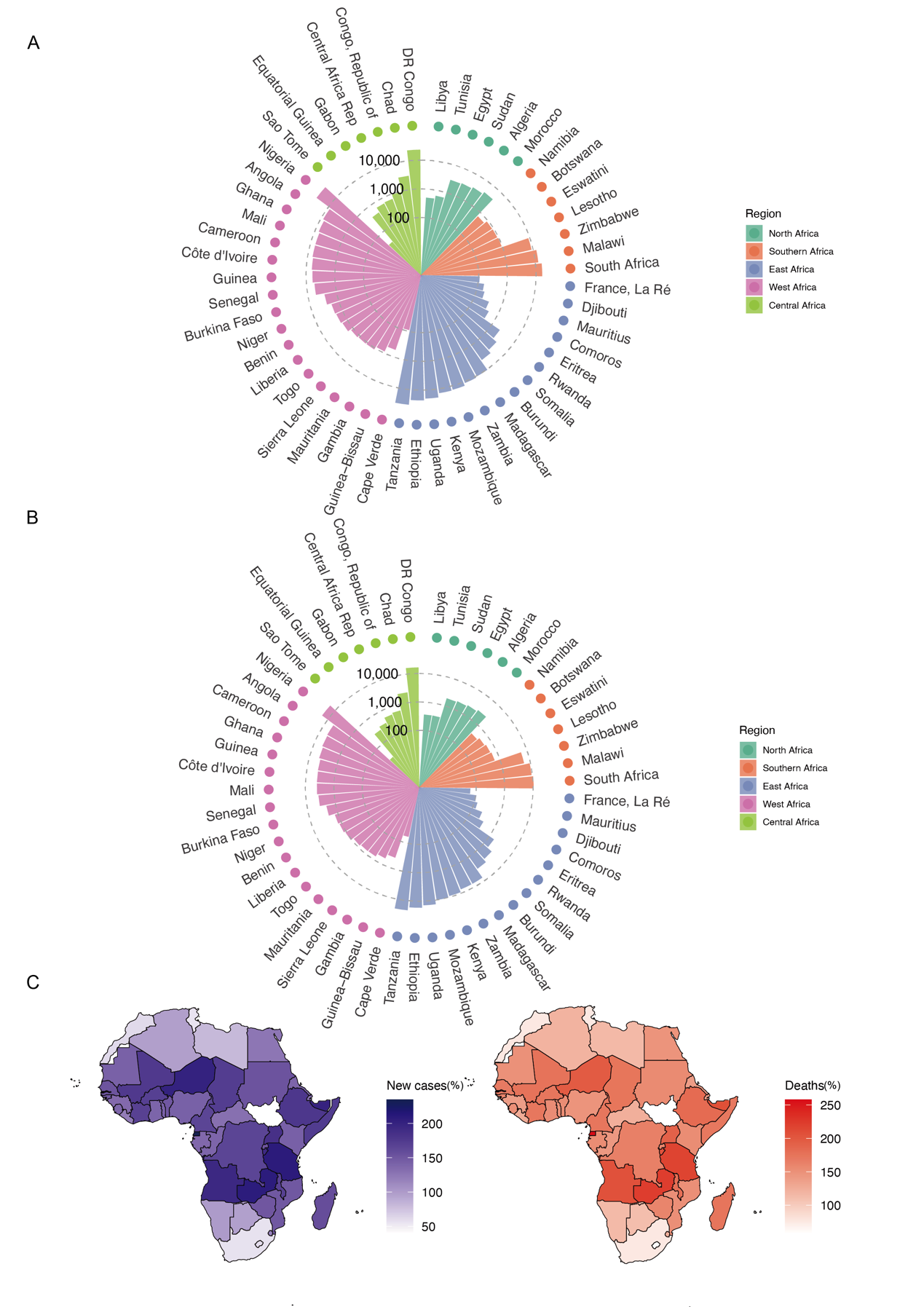


**Figure S4. Forecast of cervical cancer new cases and deaths (2050) in African countries.** The circular bar plots show the absolute change of cervical cancer new cases **(A)** and deaths **(B)** counts from 2022 to 2050, assuming no annual change in the new case or death number from the baseline year 2022. Bars are colored by region. **(C)** The maps show the percentage change of cervical cancer new cases and deaths counts from 2022 to 2050.

**Table S1. Cervical cancer incidence and mortality in Africa and World in 2022 by age.**

| **Location** | **ASIR** | **New cases** | **ASMR** | **Deaths** | **Age** |
| --- | --- | --- | --- | --- | --- |
| Africa | 2.58 | 4527 | 1.00 | 1765 | 15 to 29 |
| Africa | 26.09 | 30873 | 12.79 | 15014 | 30 to 44 |
| Africa | 65.37 | 45938 | 40.81 | 28672 | 45 to 59 |
| Africa | 98.36 | 33621 | 75.01 | 25659 | 60 to 74 |
| Africa | 120.45 | 10702 | 107.56 | 9502 | 75 to 85+ |
| World | 2.26 | 19938 | 0.68 | 4616 | 15 to 29 |
| World | 19.24 | 153931 | 7.91 | 54344 | 30 to 44 |
| World | 39.02 | 262476 | 22.11 | 124174 | 45 to 59 |
| World | 40.26 | 170217 | 35.97 | 113158 | 60 to 74 |
| World | 31.93 | 55632 | 43.92 | 52556 | 75 to 85+ |

**Table S2. Peak age and age-standardized rates of cervical cancer incidence and mortality in Africa in 2022.**

| **Location** | **ASIR** | **New cases** | **Peak age** | **ASMR** | **Deaths** | **Peak age** | **Region** |
| --- | --- | --- | --- | --- | --- | --- | --- |
| Sudan | 62.67 | 184 | 75 to 85+ | 47.99 | 140 | 75 to 85+ | North Africa |
| Morocco | 46.23 | 883 | 60 to 74 | 33.56 | 641 | 60 to 74 | North Africa |
| Algeria | 34.5 | 607 | 60 to 74 | 24.7 | 436 | 60 to 74 | North Africa |
| Libya | 29.72 | 177 | 45 to 59 | 36.55 | 23 | 75 to 85+ | North Africa |
| Egypt | 23.51 | 251 | 75 to 85+ | 22.01 | 235 | 75 to 85+ | North Africa |
| Tunisia | 18.61 | 129 | 60 to 74 | 12.67 | 88 | 60 to 74 | North Africa |
| Zimbabwe | 300.42 | 313 | 75 to 85+ | 290.21 | 301 | 75 to 85+ | Southern Africa |
| Eswatini | 272.76 | 136 | 45 to 59 | 269.64 | 33 | 75 to 85+ | Southern Africa |
| Lesotho | 211.72 | 53 | 75 to 85+ | 211.72 | 53 | 75 to 85+ | Southern Africa |
| Malawi | 188.7 | 1589 | 45 to 59 | 174.41 | 660 | 60 to 74 | Southern Africa |
| Namibia | 179.83 | 37 | 75 to 85+ | 170.42 | 35 | 75 to 85+ | Southern Africa |
| Botswana | 145.1 | 32 | 75 to 85+ | 133.14 | 29 | 75 to 85+ | Southern Africa |
| South Africa | 91.58 | 4066 | 45 to 59 | 120.34 | 805 | 75 to 85+ | Southern Africa |
| Tanzania | 547.58 | 1564 | 75 to 85+ | 456.12 | 1288 | 75 to 85+ | East Africa |
| Zambia | 400.55 | 324 | 75 to 85+ | 399.08 | 315 | 75 to 85+ | East Africa |
| Comoros | 257.71 | 12 | 75 to 85+ | 168.1 | 8 | 75 to 85+ | East Africa |
| Madagascar | 215.34 | 316 | 75 to 85+ | 218.18 | 318 | 75 to 85+ | East Africa |
| Burundi | 214.41 | 100 | 75 to 85+ | 214.41 | 100 | 75 to 85+ | East Africa |
| Uganda | 177.52 | 1343 | 60 to 74 | 163.5 | 1237 | 60 to 74 | East Africa |
| Kenya | 164.12 | 367 | 75 to 85+ | 116.92 | 285 | 75 to 85+ | East Africa |
| Mozambique | 117.81 | 1720 | 45 to 59 | 105.35 | 725 | 60 to 74 | East Africa |
| Somalia | 98.46 | 314 | 60 to 74 | 96.59 | 74 | 75 to 85+ | East Africa |
| South Sudan | 91.3 | 65 | 75 to 85+ | 92.55 | 66 | 75 to 85+ | East Africa |
| Ethiopia | 88.04 | 2388 | 60 to 74 | 76.97 | 2095 | 60 to 74 | East Africa |
| Mauritius | 86.18 | 31 | 75 to 85+ | 61.12 | 22 | 75 to 85+ | East Africa |
| Eritrea | 72.3 | 24 | 75 to 85+ | 72.3 | 24 | 75 to 85+ | East Africa |
| Djibouti | 69.91 | 22 | 60 to 74 | 63.62 | 20 | 60 to 74 | East Africa |
| Rwanda | 67.13 | 227 | 60 to 74 | 58.05 | 196 | 60 to 74 | East Africa |
| France, La Ré | 39.23 | 11 | 75 to 85+ | 25.95 | 8 | 75 to 85+ | East Africa |
| Nigeria | 303.36 | 2205 | 75 to 85+ | 207.27 | 1482 | 75 to 85+ | West Africa |
| Guinea | 244.14 | 161 | 75 to 85+ | 241.69 | 159 | 75 to 85+ | West Africa |
| Cameroon | 215.77 | 260 | 75 to 85+ | 211.67 | 254 | 75 to 85+ | West Africa |
| Liberia | 181.61 | 55 | 75 to 85+ | 178.75 | 54 | 75 to 85+ | West Africa |
| Guinea-Bissau | 169.65 | 16 | 75 to 85+ | 169.65 | 16 | 75 to 85+ | West Africa |
| Côte d'Ivoire | 167.68 | 170 | 75 to 85+ | 137.37 | 139 | 75 to 85+ | West Africa |
| Mali | 165.04 | 138 | 75 to 85+ | 134.3 | 111 | 75 to 85+ | West Africa |
| Gambia | 153.02 | 14 | 75 to 85+ | 153.02 | 14 | 75 to 85+ | West Africa |
| Senegal | 148.3 | 144 | 75 to 85+ | 144.77 | 140 | 75 to 85+ | West Africa |
| Mauritania | 140.4 | 40 | 75 to 85+ | 137.31 | 39 | 75 to 85+ | West Africa |
| Angola | 126.28 | 166 | 75 to 85+ | 124.21 | 162 | 75 to 85+ | West Africa |
| Ghana | 110.11 | 878 | 60 to 74 | 95.26 | 153 | 75 to 85+ | West Africa |
| Benin | 83.71 | 236 | 60 to 74 | 66.47 | 188 | 60 to 74 | West Africa |
| Togo | 80.5 | 30 | 75 to 85+ | 78.36 | 29 | 75 to 85+ | West Africa |
| Burkina Faso | 74.76 | 319 | 60 to 74 | 65.27 | 279 | 60 to 74 | West Africa |
| Cape Verde | 67.91 | 15 | 60 to 74 | 49.83 | 3 | 75 to 85+ | West Africa |
| Sierra Leone | 43.7 | 169 | 45 to 59 | 35.46 | 62 | 60 to 74 | West Africa |
| Niger | 27.51 | 120 | 60 to 74 | 26.12 | 114 | 60 to 74 | West Africa |
| DR Congo | 176.79 | 894 | 75 to 85+ | 174.16 | 877 | 75 to 85+ | Central Africa |
| Equatorial Guinea | 170.58 | 9 | 75 to 85+ | 94.65 | 22 | 60 to 74 | Central Africa |
| Gabon | 167.35 | 27 | 75 to 85+ | 150.82 | 24 | 75 to 85+ | Central Africa |
| Central Africa Rep | 108.95 | 29 | 75 to 85+ | 108.95 | 29 | 75 to 85+ | Central Africa |
| Congo, Republic of | 98.81 | 121 | 60 to 74 | 84.32 | 23 | 75 to 85+ | Central Africa |
| Sao Tome | 81.3 | 1 | 75 to 85+ | 81.3 | 1 | 75 to 85+ | Central Africa |
| Chad | 65.65 | 406 | 45 to 59 | 54.38 | 163 | 60 to 74 | Central Africa |

ASIR, age-standardized incidence rates; ASMR age-standardized mortality rates.

**Table S3. Prediction of cervical cancer cases in Africa from 2025 to 2050.**

| **Scale** | **Type** | **2022** | **2025** | **2030** | **2035** | **2040** | **2045** | **2050** | **Percent change** |
| --- | --- | --- | --- | --- | --- | --- | --- | --- | --- |
| 3 | New cases | 125.70 | 152.06 | 207.00 | 280.70 | 378.89 | 508.95 | 680.08 | 441.04 |
| 3 | Deaths | 80.61 | 97.58 | 133.53 | 182.07 | 247.13 | 333.79 | 448.54 | 456.41 |
| 2 | New cases | 125.70 | 147.67 | 191.46 | 247.27 | 317.87 | 406.65 | 517.52 | 311.71 |
| 2 | Deaths | 80.61 | 94.76 | 123.51 | 160.38 | 207.33 | 266.70 | 341.32 | 323.40 |
| 1 | New cases | 125.70 | 143.37 | 176.95 | 217.54 | 266.22 | 324.20 | 392.75 | 212.45 |
| 1 | Deaths | 80.61 | 92.00 | 114.14 | 141.10 | 173.64 | 212.62 | 259.04 | 221.33 |
| 0 | New cases | 125.70 | 139.16 | 163.41 | 191.14 | 222.56 | 257.88 | 297.25 | 136.48 |
| 0 | Deaths | 80.61 | 89.30 | 105.41 | 123.98 | 145.16 | 169.13 | 196.05 | 143.19 |
| -1 | New cases | 125.70 | 135.02 | 150.79 | 167.73 | 185.73 | 204.66 | 224.34 | 78.47 |
| -1 | Deaths | 80.61 | 86.65 | 97.27 | 108.80 | 121.14 | 134.22 | 147.96 | 83.54 |
| -2 | New cases | 125.70 | 130.97 | 139.02 | 146.99 | 154.71 | 162.04 | 168.83 | 34.31 |
| -2 | Deaths | 80.61 | 84.05 | 89.68 | 95.34 | 100.91 | 106.27 | 111.35 | 38.13 |
| -3 | New cases | 125.70 | 127.00 | 128.07 | 128.65 | 128.63 | 127.99 | 126.69 | 0.79 |
| -3 | Deaths | 80.61 | 81.50 | 82.61 | 83.44 | 83.90 | 83.94 | 83.55 | 3.65 |

The number of cases is presented per 1000. The percentage (%) represents the change in new cases or deaths in 2050, assuming annual change (scale, %) in the new case or death number from the baseline year 2022. The data was obtained from the GLOBOCAN database in 2022.

**Table S4. Prediction of cervical cancer cases in African countries in 2050.**

| **Type** | **region** | **Population** | **scale** | **2050** | **2022** | **Percent change** |
| --- | --- | --- | --- | --- | --- | --- |
| New cases | North Africa | Algeria | 3 | 8098 | 1799 | 350.14 |
| New cases | North Africa | Algeria | 2 | 6162 | 1799 | 242.52 |
| New cases | North Africa | Algeria | 1 | 4676 | 1799 | 159.92 |
| New cases | North Africa | Algeria | 0 | 3539 | 1799 | 96.72 |
| New cases | North Africa | Algeria | -1 | 2671 | 1799 | 48.47 |
| New cases | North Africa | Algeria | -2 | 2010 | 1799 | 11.73 |
| New cases | North Africa | Algeria | -3 | 1508 | 1799 | -16.18 |
| New cases | North Africa | Egypt | 3 | 6729 | 1302 | 416.82 |
| New cases | North Africa | Egypt | 2 | 5121 | 1302 | 293.32 |
| New cases | North Africa | Egypt | 1 | 3886 | 1302 | 198.46 |
| New cases | North Africa | Egypt | 0 | 2941 | 1302 | 125.88 |
| New cases | North Africa | Egypt | -1 | 2220 | 1302 | 70.51 |
| New cases | North Africa | Egypt | -2 | 1671 | 1302 | 28.34 |
| New cases | North Africa | Egypt | -3 | 1254 | 1302 | -3.69 |
| New cases | North Africa | Libya | 3 | 1151 | 278 | 314.03 |
| New cases | North Africa | Libya | 2 | 876 | 278 | 215.11 |
| New cases | North Africa | Libya | 1 | 665 | 278 | 139.21 |
| New cases | North Africa | Libya | 0 | 503 | 278 | 80.94 |
| New cases | North Africa | Libya | -1 | 380 | 278 | 36.69 |
| New cases | North Africa | Libya | -2 | 286 | 278 | 2.88 |
| New cases | North Africa | Libya | -3 | 214 | 278 | -23.02 |
| New cases | North Africa | Morocco | 3 | 9757 | 2644 | 269.02 |
| New cases | North Africa | Morocco | 2 | 7425 | 2644 | 180.82 |
| New cases | North Africa | Morocco | 1 | 5635 | 2644 | 113.12 |
| New cases | North Africa | Morocco | 0 | 4264 | 2644 | 61.27 |
| New cases | North Africa | Morocco | -1 | 3218 | 2644 | 21.71 |
| New cases | North Africa | Morocco | -2 | 2422 | 2644 | -8.4 |
| New cases | North Africa | Morocco | -3 | 1817 | 2644 | -31.28 |
| New cases | North Africa | Sudan | 3 | 7148 | 1234 | 479.25 |
| New cases | North Africa | Sudan | 2 | 5439 | 1234 | 340.76 |
| New cases | North Africa | Sudan | 1 | 4128 | 1234 | 234.52 |
| New cases | North Africa | Sudan | 0 | 3124 | 1234 | 153.16 |
| New cases | North Africa | Sudan | -1 | 2358 | 1234 | 91.09 |
| New cases | North Africa | Sudan | -2 | 1774 | 1234 | 43.76 |
| New cases | North Africa | Sudan | -3 | 1332 | 1234 | 7.94 |
| New cases | North Africa | Tunisia | 3 | 1472 | 414 | 255.56 |
| New cases | North Africa | Tunisia | 2 | 1120 | 414 | 170.53 |
| New cases | North Africa | Tunisia | 1 | 850 | 414 | 105.31 |
| New cases | North Africa | Tunisia | 0 | 643 | 414 | 55.31 |
| New cases | North Africa | Tunisia | -1 | 485 | 414 | 17.15 |
| New cases | North Africa | Tunisia | -2 | 365 | 414 | -11.84 |
| New cases | North Africa | Tunisia | -3 | 274 | 414 | -33.82 |
| New cases | Southern Africa | Botswana | 3 | 2038 | 454 | 348.9 |
| New cases | Southern Africa | Botswana | 2 | 1551 | 454 | 241.63 |
| New cases | Southern Africa | Botswana | 1 | 1177 | 454 | 159.25 |
| New cases | Southern Africa | Botswana | 0 | 891 | 454 | 96.26 |
| New cases | Southern Africa | Botswana | -1 | 672 | 454 | 48.02 |
| New cases | Southern Africa | Botswana | -2 | 506 | 454 | 11.45 |
| New cases | Southern Africa | Botswana | -3 | 380 | 454 | -16.3 |
| New cases | Southern Africa | Eswatini | 3 | 2075 | 417 | 397.6 |
| New cases | Southern Africa | Eswatini | 2 | 1579 | 417 | 278.66 |
| New cases | Southern Africa | Eswatini | 1 | 1198 | 417 | 187.29 |
| New cases | Southern Africa | Eswatini | 0 | 907 | 417 | 117.51 |
| New cases | Southern Africa | Eswatini | -1 | 684 | 417 | 64.03 |
| New cases | Southern Africa | Eswatini | -2 | 515 | 417 | 23.5 |
| New cases | Southern Africa | Eswatini | -3 | 386 | 417 | -7.43 |
| New cases | Southern Africa | Lesotho | 3 | 2173 | 598 | 263.38 |
| New cases | Southern Africa | Lesotho | 2 | 1654 | 598 | 176.59 |
| New cases | Southern Africa | Lesotho | 1 | 1255 | 598 | 109.87 |
| New cases | Southern Africa | Lesotho | 0 | 950 | 598 | 58.86 |
| New cases | Southern Africa | Lesotho | -1 | 717 | 598 | 19.9 |
| New cases | Southern Africa | Lesotho | -2 | 539 | 598 | -9.87 |
| New cases | Southern Africa | Lesotho | -3 | 405 | 598 | -32.27 |
| New cases | Southern Africa | Malawi | 3 | 29030 | 4701 | 517.53 |
| New cases | Southern Africa | Malawi | 2 | 22090 | 4701 | 369.9 |
| New cases | Southern Africa | Malawi | 1 | 16765 | 4701 | 256.63 |
| New cases | Southern Africa | Malawi | 0 | 12688 | 4701 | 169.9 |
| New cases | Southern Africa | Malawi | -1 | 9576 | 4701 | 103.7 |
| New cases | Southern Africa | Malawi | -2 | 7207 | 4701 | 53.31 |
| New cases | Southern Africa | Malawi | -3 | 5408 | 4701 | 15.04 |
| New cases | Southern Africa | Namibia | 3 | 1604 | 350 | 358.29 |
| New cases | Southern Africa | Namibia | 2 | 1221 | 350 | 248.86 |
| New cases | Southern Africa | Namibia | 1 | 926 | 350 | 164.57 |
| New cases | Southern Africa | Namibia | 0 | 701 | 350 | 100.29 |
| New cases | Southern Africa | Namibia | -1 | 529 | 350 | 51.14 |
| New cases | Southern Africa | Namibia | -2 | 398 | 350 | 13.71 |
| New cases | Southern Africa | Namibia | -3 | 299 | 350 | -14.57 |
| New cases | Southern Africa | South Africa | 3 | 37633 | 10532 | 257.32 |
| New cases | Southern Africa | South Africa | 2 | 28637 | 10532 | 171.9 |
| New cases | Southern Africa | South Africa | 1 | 21733 | 10532 | 106.35 |
| New cases | Southern Africa | South Africa | 0 | 16449 | 10532 | 56.18 |
| New cases | Southern Africa | South Africa | -1 | 12414 | 10532 | 17.87 |
| New cases | Southern Africa | South Africa | -2 | 9342 | 10532 | -11.3 |
| New cases | Southern Africa | South Africa | -3 | 7010 | 10532 | -33.44 |
| New cases | Southern Africa | Zimbabwe | 3 | 20195 | 3520 | 473.72 |
| New cases | Southern Africa | Zimbabwe | 2 | 15367 | 3520 | 336.56 |
| New cases | Southern Africa | Zimbabwe | 1 | 11663 | 3520 | 231.34 |
| New cases | Southern Africa | Zimbabwe | 0 | 8827 | 3520 | 150.77 |
| New cases | Southern Africa | Zimbabwe | -1 | 6662 | 3520 | 89.26 |
| New cases | Southern Africa | Zimbabwe | -2 | 5013 | 3520 | 42.41 |
| New cases | Southern Africa | Zimbabwe | -3 | 3762 | 3520 | 6.88 |
| New cases | East Africa | Burundi | 3 | 9771 | 1457 | 570.62 |
| New cases | East Africa | Burundi | 2 | 7436 | 1457 | 410.36 |
| New cases | East Africa | Burundi | 1 | 5643 | 1457 | 287.3 |
| New cases | East Africa | Burundi | 0 | 4271 | 1457 | 193.14 |
| New cases | East Africa | Burundi | -1 | 3223 | 1457 | 121.21 |
| New cases | East Africa | Burundi | -2 | 2426 | 1457 | 66.51 |
| New cases | East Africa | Burundi | -3 | 1820 | 1457 | 24.91 |
| New cases | East Africa | Comoros | 3 | 785 | 163 | 381.6 |
| New cases | East Africa | Comoros | 2 | 597 | 163 | 266.26 |
| New cases | East Africa | Comoros | 1 | 453 | 163 | 177.91 |
| New cases | East Africa | Comoros | 0 | 343 | 163 | 110.43 |
| New cases | East Africa | Comoros | -1 | 259 | 163 | 58.9 |
| New cases | East Africa | Comoros | -2 | 195 | 163 | 19.63 |
| New cases | East Africa | Comoros | -3 | 146 | 163 | -10.43 |
| New cases | East Africa | Djibouti | 3 | 373 | 71 | 425.35 |
| New cases | East Africa | Djibouti | 2 | 284 | 71 | 300 |
| New cases | East Africa | Djibouti | 1 | 215 | 71 | 202.82 |
| New cases | East Africa | Djibouti | 0 | 163 | 71 | 129.58 |
| New cases | East Africa | Djibouti | -1 | 123 | 71 | 73.24 |
| New cases | East Africa | Djibouti | -2 | 93 | 71 | 30.99 |
| New cases | East Africa | Djibouti | -3 | 69 | 71 | -2.82 |
| New cases | East Africa | Eritrea | 3 | 1068 | 196 | 444.9 |
| New cases | East Africa | Eritrea | 2 | 813 | 196 | 314.8 |
| New cases | East Africa | Eritrea | 1 | 617 | 196 | 214.8 |
| New cases | East Africa | Eritrea | 0 | 467 | 196 | 138.27 |
| New cases | East Africa | Eritrea | -1 | 352 | 196 | 79.59 |
| New cases | East Africa | Eritrea | -2 | 265 | 196 | 35.2 |
| New cases | East Africa | Eritrea | -3 | 199 | 196 | 1.53 |
| New cases | East Africa | Ethiopia | 3 | 51918 | 8168 | 535.63 |
| New cases | East Africa | Ethiopia | 2 | 39508 | 8168 | 383.69 |
| New cases | East Africa | Ethiopia | 1 | 29983 | 8168 | 267.08 |
| New cases | East Africa | Ethiopia | 0 | 22692 | 8168 | 177.82 |
| New cases | East Africa | Ethiopia | -1 | 17126 | 8168 | 109.67 |
| New cases | East Africa | Ethiopia | -2 | 12889 | 8168 | 57.8 |
| New cases | East Africa | Ethiopia | -3 | 9671 | 8168 | 18.4 |
| New cases | East Africa | France, La Ré | 3 | 249 | 75 | 232 |
| New cases | East Africa | France, La Ré | 2 | 189 | 75 | 152 |
| New cases | East Africa | France, La Ré | 1 | 144 | 75 | 92 |
| New cases | East Africa | France, La Ré | 0 | 109 | 75 | 45.33 |
| New cases | East Africa | France, La Ré | -1 | 82 | 75 | 9.33 |
| New cases | East Africa | France, La Ré | -2 | 62 | 75 | -17.33 |
| New cases | East Africa | France, La Ré | -3 | 46 | 75 | -38.67 |
| New cases | East Africa | Kenya | 3 | 32425 | 5845 | 454.75 |
| New cases | East Africa | Kenya | 2 | 24674 | 5845 | 322.14 |
| New cases | East Africa | Kenya | 1 | 18725 | 5845 | 220.36 |
| New cases | East Africa | Kenya | 0 | 14172 | 5845 | 142.46 |
| New cases | East Africa | Kenya | -1 | 10696 | 5845 | 82.99 |
| New cases | East Africa | Kenya | -2 | 8049 | 5845 | 37.71 |
| New cases | East Africa | Kenya | -3 | 6040 | 5845 | 3.34 |
| New cases | East Africa | Madagascar | 3 | 23881 | 4060 | 488.2 |
| New cases | East Africa | Madagascar | 2 | 18173 | 4060 | 347.61 |
| New cases | East Africa | Madagascar | 1 | 13791 | 4060 | 239.68 |
| New cases | East Africa | Madagascar | 0 | 10438 | 4060 | 157.09 |
| New cases | East Africa | Madagascar | -1 | 7878 | 4060 | 94.04 |
| New cases | East Africa | Madagascar | -2 | 5928 | 4060 | 46.01 |
| New cases | East Africa | Madagascar | -3 | 4449 | 4060 | 9.58 |
| New cases | East Africa | Mauritius | 3 | 434 | 136 | 219.12 |
| New cases | East Africa | Mauritius | 2 | 331 | 136 | 143.38 |
| New cases | East Africa | Mauritius | 1 | 251 | 136 | 84.56 |
| New cases | East Africa | Mauritius | 0 | 190 | 136 | 39.71 |
| New cases | East Africa | Mauritius | -1 | 143 | 136 | 5.15 |
| New cases | East Africa | Mauritius | -2 | 108 | 136 | -20.59 |
| New cases | East Africa | Mauritius | -3 | 81 | 136 | -40.44 |
| New cases | East Africa | Mozambique | 3 | 31135 | 5456 | 470.66 |
| New cases | East Africa | Mozambique | 2 | 23692 | 5456 | 334.24 |
| New cases | East Africa | Mozambique | 1 | 17981 | 5456 | 229.56 |
| New cases | East Africa | Mozambique | 0 | 13608 | 5456 | 149.41 |
| New cases | East Africa | Mozambique | -1 | 10270 | 5456 | 88.23 |
| New cases | East Africa | Mozambique | -2 | 7729 | 5456 | 41.66 |
| New cases | East Africa | Mozambique | -3 | 5800 | 5456 | 6.3 |
| New cases | East Africa | Rwanda | 3 | 4983 | 866 | 475.4 |
| New cases | East Africa | Rwanda | 2 | 3792 | 866 | 337.88 |
| New cases | East Africa | Rwanda | 1 | 2878 | 866 | 232.33 |
| New cases | East Africa | Rwanda | 0 | 2178 | 866 | 151.5 |
| New cases | East Africa | Rwanda | -1 | 1644 | 866 | 89.84 |
| New cases | East Africa | Rwanda | -2 | 1237 | 866 | 42.84 |
| New cases | East Africa | Rwanda | -3 | 928 | 866 | 7.16 |
| New cases | East Africa | Somalia | 3 | 7311 | 1167 | 526.48 |
| New cases | East Africa | Somalia | 2 | 5564 | 1167 | 376.78 |
| New cases | East Africa | Somalia | 1 | 4222 | 1167 | 261.78 |
| New cases | East Africa | Somalia | 0 | 3196 | 1167 | 173.86 |
| New cases | East Africa | Somalia | -1 | 2412 | 1167 | 106.68 |
| New cases | East Africa | Somalia | -2 | 1815 | 1167 | 55.53 |
| New cases | East Africa | Somalia | -3 | 1362 | 1167 | 16.71 |
| New cases | East Africa | Tanzania | 3 | 76619 | 10868 | 605 |
| New cases | East Africa | Tanzania | 2 | 58304 | 10868 | 436.47 |
| New cases | East Africa | Tanzania | 1 | 44248 | 10868 | 307.14 |
| New cases | East Africa | Tanzania | 0 | 33488 | 10868 | 208.13 |
| New cases | East Africa | Tanzania | -1 | 25274 | 10868 | 132.55 |
| New cases | East Africa | Tanzania | -2 | 19021 | 10868 | 75.02 |
| New cases | East Africa | Tanzania | -3 | 14273 | 10868 | 31.33 |
| New cases | East Africa | Uganda | 3 | 45084 | 6938 | 549.81 |
| New cases | East Africa | Uganda | 2 | 34307 | 6938 | 394.48 |
| New cases | East Africa | Uganda | 1 | 26036 | 6938 | 275.27 |
| New cases | East Africa | Uganda | 0 | 19705 | 6938 | 184.02 |
| New cases | East Africa | Uganda | -1 | 14872 | 6938 | 114.36 |
| New cases | East Africa | Uganda | -2 | 11192 | 6938 | 61.31 |
| New cases | East Africa | Uganda | -3 | 8398 | 6938 | 21.04 |
| New cases | East Africa | Zambia | 3 | 25636 | 3640 | 604.29 |
| New cases | East Africa | Zambia | 2 | 19508 | 3640 | 435.93 |
| New cases | East Africa | Zambia | 1 | 14805 | 3640 | 306.73 |
| New cases | East Africa | Zambia | 0 | 11205 | 3640 | 207.83 |
| New cases | East Africa | Zambia | -1 | 8456 | 3640 | 132.31 |
| New cases | East Africa | Zambia | -2 | 6364 | 3640 | 74.84 |
| New cases | East Africa | Zambia | -3 | 4775 | 3640 | 31.18 |
| New cases | West Africa | Angola | 3 | 19021 | 2823 | 573.79 |
| New cases | West Africa | Angola | 2 | 14474 | 2823 | 412.72 |
| New cases | West Africa | Angola | 1 | 10985 | 2823 | 289.13 |
| New cases | West Africa | Angola | 0 | 8314 | 2823 | 194.51 |
| New cases | West Africa | Angola | -1 | 6274 | 2823 | 122.25 |
| New cases | West Africa | Angola | -2 | 4722 | 2823 | 67.27 |
| New cases | West Africa | Angola | -3 | 3543 | 2823 | 25.5 |
| New cases | West Africa | Benin | 3 | 4011 | 701 | 472.18 |
| New cases | West Africa | Benin | 2 | 3052 | 701 | 335.38 |
| New cases | West Africa | Benin | 1 | 2316 | 701 | 230.39 |
| New cases | West Africa | Benin | 0 | 1753 | 701 | 150.07 |
| New cases | West Africa | Benin | -1 | 1323 | 701 | 88.73 |
| New cases | West Africa | Benin | -2 | 996 | 701 | 42.08 |
| New cases | West Africa | Benin | -3 | 747 | 701 | 6.56 |
| New cases | West Africa | Burkina Faso | 3 | 6097 | 988 | 517.11 |
| New cases | West Africa | Burkina Faso | 2 | 4640 | 988 | 369.64 |
| New cases | West Africa | Burkina Faso | 1 | 3521 | 988 | 256.38 |
| New cases | West Africa | Burkina Faso | 0 | 2665 | 988 | 169.74 |
| New cases | West Africa | Burkina Faso | -1 | 2011 | 988 | 103.54 |
| New cases | West Africa | Burkina Faso | -2 | 1514 | 988 | 53.24 |
| New cases | West Africa | Burkina Faso | -3 | 1136 | 988 | 14.98 |
| New cases | West Africa | Cameroon | 3 | 15331 | 2525 | 507.17 |
| New cases | West Africa | Cameroon | 2 | 11666 | 2525 | 362.02 |
| New cases | West Africa | Cameroon | 1 | 8854 | 2525 | 250.65 |
| New cases | West Africa | Cameroon | 0 | 6701 | 2525 | 165.39 |
| New cases | West Africa | Cameroon | -1 | 5057 | 2525 | 100.28 |
| New cases | West Africa | Cameroon | -2 | 3806 | 2525 | 50.73 |
| New cases | West Africa | Cameroon | -3 | 2856 | 2525 | 13.11 |
| New cases | West Africa | Cape Verde | 3 | 203 | 46 | 341.3 |
| New cases | West Africa | Cape Verde | 2 | 155 | 46 | 236.96 |
| New cases | West Africa | Cape Verde | 1 | 117 | 46 | 154.35 |
| New cases | West Africa | Cape Verde | 0 | 89 | 46 | 93.48 |
| New cases | West Africa | Cape Verde | -1 | 67 | 46 | 45.65 |
| New cases | West Africa | Cape Verde | -2 | 50 | 46 | 8.7 |
| New cases | West Africa | Cape Verde | -3 | 38 | 46 | -17.39 |
| New cases | West Africa | Côte d'Ivoire | 3 | 14523 | 2360 | 515.38 |
| New cases | West Africa | Côte d'Ivoire | 2 | 11052 | 2360 | 368.31 |
| New cases | West Africa | Côte d'Ivoire | 1 | 8387 | 2360 | 255.38 |
| New cases | West Africa | Côte d'Ivoire | 0 | 6348 | 2360 | 168.98 |
| New cases | West Africa | Côte d'Ivoire | -1 | 4791 | 2360 | 103.01 |
| New cases | West Africa | Côte d'Ivoire | -2 | 3605 | 2360 | 52.75 |
| New cases | West Africa | Côte d'Ivoire | -3 | 2705 | 2360 | 14.62 |
| New cases | West Africa | Gambia | 3 | 1998 | 325 | 514.77 |
| New cases | West Africa | Gambia | 2 | 1520 | 325 | 367.69 |
| New cases | West Africa | Gambia | 1 | 1154 | 325 | 255.08 |
| New cases | West Africa | Gambia | 0 | 873 | 325 | 168.62 |
| New cases | West Africa | Gambia | -1 | 659 | 325 | 102.77 |
| New cases | West Africa | Gambia | -2 | 496 | 325 | 52.62 |
| New cases | West Africa | Gambia | -3 | 372 | 325 | 14.46 |
| New cases | West Africa | Ghana | 3 | 16816 | 3072 | 447.4 |
| New cases | West Africa | Ghana | 2 | 12797 | 3072 | 316.57 |
| New cases | West Africa | Ghana | 1 | 9712 | 3072 | 216.15 |
| New cases | West Africa | Ghana | 0 | 7350 | 3072 | 139.26 |
| New cases | West Africa | Ghana | -1 | 5547 | 3072 | 80.57 |
| New cases | West Africa | Ghana | -2 | 4175 | 3072 | 35.9 |
| New cases | West Africa | Ghana | -3 | 3133 | 3072 | 1.99 |
| New cases | West Africa | Guinea | 3 | 13707 | 2551 | 437.32 |
| New cases | West Africa | Guinea | 2 | 10430 | 2551 | 308.86 |
| New cases | West Africa | Guinea | 1 | 7916 | 2551 | 210.31 |
| New cases | West Africa | Guinea | 0 | 5991 | 2551 | 134.85 |
| New cases | West Africa | Guinea | -1 | 4521 | 2551 | 77.22 |
| New cases | West Africa | Guinea | -2 | 3403 | 2551 | 33.4 |
| New cases | West Africa | Guinea | -3 | 2553 | 2551 | 0.08 |
| New cases | West Africa | Guinea-Bissau | 3 | 1275 | 224 | 469.2 |
| New cases | West Africa | Guinea-Bissau | 2 | 971 | 224 | 333.48 |
| New cases | West Africa | Guinea-Bissau | 1 | 737 | 224 | 229.02 |
| New cases | West Africa | Guinea-Bissau | 0 | 557 | 224 | 148.66 |
| New cases | West Africa | Guinea-Bissau | -1 | 421 | 224 | 87.95 |
| New cases | West Africa | Guinea-Bissau | -2 | 317 | 224 | 41.52 |
| New cases | West Africa | Guinea-Bissau | -3 | 238 | 224 | 6.25 |
| New cases | West Africa | Liberia | 3 | 3627 | 717 | 405.86 |
| New cases | West Africa | Liberia | 2 | 2760 | 717 | 284.94 |
| New cases | West Africa | Liberia | 1 | 2095 | 717 | 192.19 |
| New cases | West Africa | Liberia | 0 | 1585 | 717 | 121.06 |
| New cases | West Africa | Liberia | -1 | 1196 | 717 | 66.81 |
| New cases | West Africa | Liberia | -2 | 900 | 717 | 25.52 |
| New cases | West Africa | Liberia | -3 | 676 | 717 | -5.72 |
| New cases | West Africa | Mali | 3 | 15374 | 2436 | 531.12 |
| New cases | West Africa | Mali | 2 | 11699 | 2436 | 380.25 |
| New cases | West Africa | Mali | 1 | 8878 | 2436 | 264.45 |
| New cases | West Africa | Mali | 0 | 6719 | 2436 | 175.82 |
| New cases | West Africa | Mali | -1 | 5071 | 2436 | 108.17 |
| New cases | West Africa | Mali | -2 | 3816 | 2436 | 56.65 |
| New cases | West Africa | Mali | -3 | 2864 | 2436 | 17.57 |
| New cases | West Africa | Mauritania | 3 | 2593 | 468 | 454.06 |
| New cases | West Africa | Mauritania | 2 | 1973 | 468 | 321.58 |
| New cases | West Africa | Mauritania | 1 | 1497 | 468 | 219.87 |
| New cases | West Africa | Mauritania | 0 | 1133 | 468 | 142.09 |
| New cases | West Africa | Mauritania | -1 | 855 | 468 | 82.69 |
| New cases | West Africa | Mauritania | -2 | 644 | 468 | 37.61 |
| New cases | West Africa | Mauritania | -3 | 483 | 468 | 3.21 |
| New cases | West Africa | Niger | 3 | 4300 | 624 | 589.1 |
| New cases | West Africa | Niger | 2 | 3272 | 624 | 424.36 |
| New cases | West Africa | Niger | 1 | 2483 | 624 | 297.92 |
| New cases | West Africa | Niger | 0 | 1879 | 624 | 201.12 |
| New cases | West Africa | Niger | -1 | 1418 | 624 | 127.24 |
| New cases | West Africa | Niger | -2 | 1067 | 624 | 70.99 |
| New cases | West Africa | Niger | -3 | 801 | 624 | 28.37 |
| New cases | West Africa | Nigeria | 3 | 75818 | 13676 | 454.39 |
| New cases | West Africa | Nigeria | 2 | 57695 | 13676 | 321.87 |
| New cases | West Africa | Nigeria | 1 | 43785 | 13676 | 220.16 |
| New cases | West Africa | Nigeria | 0 | 33138 | 13676 | 142.31 |
| New cases | West Africa | Nigeria | -1 | 25010 | 13676 | 82.88 |
| New cases | West Africa | Nigeria | -2 | 18822 | 13676 | 37.63 |
| New cases | West Africa | Nigeria | -3 | 14123 | 13676 | 3.27 |
| New cases | West Africa | Senegal | 3 | 11937 | 2064 | 478.34 |
| New cases | West Africa | Senegal | 2 | 9083 | 2064 | 340.07 |
| New cases | West Africa | Senegal | 1 | 6894 | 2064 | 234.01 |
| New cases | West Africa | Senegal | 0 | 5217 | 2064 | 152.76 |
| New cases | West Africa | Senegal | -1 | 3938 | 2064 | 90.79 |
| New cases | West Africa | Senegal | -2 | 2963 | 2064 | 43.56 |
| New cases | West Africa | Senegal | -3 | 2224 | 2064 | 7.75 |
| New cases | West Africa | Sierra Leone | 3 | 2641 | 486 | 443.42 |
| New cases | West Africa | Sierra Leone | 2 | 2009 | 486 | 313.37 |
| New cases | West Africa | Sierra Leone | 1 | 1525 | 486 | 213.79 |
| New cases | West Africa | Sierra Leone | 0 | 1154 | 486 | 137.45 |
| New cases | West Africa | Sierra Leone | -1 | 871 | 486 | 79.22 |
| New cases | West Africa | Sierra Leone | -2 | 656 | 486 | 34.98 |
| New cases | West Africa | Sierra Leone | -3 | 492 | 486 | 1.23 |
| New cases | West Africa | Togo | 3 | 2823 | 511 | 452.45 |
| New cases | West Africa | Togo | 2 | 2149 | 511 | 320.55 |
| New cases | West Africa | Togo | 1 | 1631 | 511 | 219.18 |
| New cases | West Africa | Togo | 0 | 1234 | 511 | 141.49 |
| New cases | West Africa | Togo | -1 | 931 | 511 | 82.19 |
| New cases | West Africa | Togo | -2 | 701 | 511 | 37.18 |
| New cases | West Africa | Togo | -3 | 526 | 511 | 2.94 |
| New cases | Central Africa | Central Africa Rep | 3 | 1554 | 295 | 426.78 |
| New cases | Central Africa | Central Africa Rep | 2 | 1182 | 295 | 300.68 |
| New cases | Central Africa | Central Africa Rep | 1 | 897 | 295 | 204.07 |
| New cases | Central Africa | Central Africa Rep | 0 | 679 | 295 | 130.17 |
| New cases | Central Africa | Central Africa Rep | -1 | 513 | 295 | 73.9 |
| New cases | Central Africa | Central Africa Rep | -2 | 386 | 295 | 30.85 |
| New cases | Central Africa | Central Africa Rep | -3 | 289 | 295 | -2.03 |
| New cases | Central Africa | Chad | 3 | 6939 | 1111 | 524.57 |
| New cases | Central Africa | Chad | 2 | 5280 | 1111 | 375.25 |
| New cases | Central Africa | Chad | 1 | 4007 | 1111 | 260.67 |
| New cases | Central Africa | Chad | 0 | 3033 | 1111 | 173 |
| New cases | Central Africa | Chad | -1 | 2289 | 1111 | 106.03 |
| New cases | Central Africa | Chad | -2 | 1722 | 1111 | 55 |
| New cases | Central Africa | Chad | -3 | 1293 | 1111 | 16.38 |
| New cases | Central Africa | Congo, Republic of | 3 | 2163 | 397 | 444.84 |
| New cases | Central Africa | Congo, Republic of | 2 | 1646 | 397 | 314.61 |
| New cases | Central Africa | Congo, Republic of | 1 | 1249 | 397 | 214.61 |
| New cases | Central Africa | Congo, Republic of | 0 | 945 | 397 | 138.04 |
| New cases | Central Africa | Congo, Republic of | -1 | 713 | 397 | 79.6 |
| New cases | Central Africa | Congo, Republic of | -2 | 537 | 397 | 35.26 |
| New cases | Central Africa | Congo, Republic of | -3 | 403 | 397 | 1.51 |
| New cases | Central Africa | DR Congo | 3 | 52847 | 8705 | 507.09 |
| New cases | Central Africa | DR Congo | 2 | 40215 | 8705 | 361.98 |
| New cases | Central Africa | DR Congo | 1 | 30519 | 8705 | 250.59 |
| New cases | Central Africa | DR Congo | 0 | 23098 | 8705 | 165.34 |
| New cases | Central Africa | DR Congo | -1 | 17433 | 8705 | 100.26 |
| New cases | Central Africa | DR Congo | -2 | 13119 | 8705 | 50.71 |
| New cases | Central Africa | DR Congo | -3 | 9844 | 8705 | 13.08 |
| New cases | Central Africa | Equatorial Guinea | 3 | 973 | 127 | 666.14 |
| New cases | Central Africa | Equatorial Guinea | 2 | 741 | 127 | 483.46 |
| New cases | Central Africa | Equatorial Guinea | 1 | 562 | 127 | 342.52 |
| New cases | Central Africa | Equatorial Guinea | 0 | 425 | 127 | 234.65 |
| New cases | Central Africa | Equatorial Guinea | -1 | 321 | 127 | 152.76 |
| New cases | Central Africa | Equatorial Guinea | -2 | 242 | 127 | 90.55 |
| New cases | Central Africa | Equatorial Guinea | -3 | 181 | 127 | 42.52 |
| New cases | Central Africa | Gabon | 3 | 1461 | 271 | 439.11 |
| New cases | Central Africa | Gabon | 2 | 1112 | 271 | 310.33 |
| New cases | Central Africa | Gabon | 1 | 844 | 271 | 211.44 |
| New cases | Central Africa | Gabon | 0 | 639 | 271 | 135.79 |
| New cases | Central Africa | Gabon | -1 | 482 | 271 | 77.86 |
| New cases | Central Africa | Gabon | -2 | 363 | 271 | 33.95 |
| New cases | Central Africa | Gabon | -3 | 272 | 271 | 0.37 |
| New cases | Central Africa | Sao Tome | 3 | 76 | 14 | 442.86 |
| New cases | Central Africa | Sao Tome | 2 | 58 | 14 | 314.29 |
| New cases | Central Africa | Sao Tome | 1 | 44 | 14 | 214.29 |
| New cases | Central Africa | Sao Tome | 0 | 33 | 14 | 135.71 |
| New cases | Central Africa | Sao Tome | -1 | 25 | 14 | 78.57 |
| New cases | Central Africa | Sao Tome | -2 | 19 | 14 | 35.71 |
| New cases | Central Africa | Sao Tome | -3 | 14 | 14 | 0 |
| Deaths | North Africa | Algeria | 3 | 5111 | 1013 | 404.54 |
| Deaths | North Africa | Algeria | 2 | 3889 | 1013 | 283.91 |
| Deaths | North Africa | Algeria | 1 | 2951 | 1013 | 191.31 |
| Deaths | North Africa | Algeria | 0 | 2234 | 1013 | 120.53 |
| Deaths | North Africa | Algeria | -1 | 1686 | 1013 | 66.44 |
| Deaths | North Africa | Algeria | -2 | 1269 | 1013 | 25.27 |
| Deaths | North Africa | Algeria | -3 | 952 | 1013 | -6.02 |
| Deaths | North Africa | Egypt | 3 | 4646 | 820 | 466.59 |
| Deaths | North Africa | Egypt | 2 | 3535 | 820 | 331.1 |
| Deaths | North Africa | Egypt | 1 | 2683 | 820 | 227.2 |
| Deaths | North Africa | Egypt | 0 | 2031 | 820 | 147.68 |
| Deaths | North Africa | Egypt | -1 | 1533 | 820 | 86.95 |
| Deaths | North Africa | Egypt | -2 | 1153 | 820 | 40.61 |
| Deaths | North Africa | Egypt | -3 | 865 | 820 | 5.49 |
| Deaths | North Africa | Libya | 3 | 835 | 169 | 394.08 |
| Deaths | North Africa | Libya | 2 | 635 | 169 | 275.74 |
| Deaths | North Africa | Libya | 1 | 482 | 169 | 185.21 |
| Deaths | North Africa | Libya | 0 | 365 | 169 | 115.98 |
| Deaths | North Africa | Libya | -1 | 275 | 169 | 62.72 |
| Deaths | North Africa | Libya | -2 | 207 | 169 | 22.49 |
| Deaths | North Africa | Libya | -3 | 155 | 169 | -8.28 |
| Deaths | North Africa | Morocco | 3 | 5898 | 1468 | 301.77 |
| Deaths | North Africa | Morocco | 2 | 4488 | 1468 | 205.72 |
| Deaths | North Africa | Morocco | 1 | 3406 | 1468 | 132.02 |
| Deaths | North Africa | Morocco | 0 | 2578 | 1468 | 75.61 |
| Deaths | North Africa | Morocco | -1 | 1946 | 1468 | 32.56 |
| Deaths | North Africa | Morocco | -2 | 1464 | 1468 | -0.27 |
| Deaths | North Africa | Morocco | -3 | 1099 | 1468 | -25.14 |
| Deaths | North Africa | Sudan | 3 | 4306 | 738 | 483.47 |
| Deaths | North Africa | Sudan | 2 | 3276 | 738 | 343.9 |
| Deaths | North Africa | Sudan | 1 | 2487 | 738 | 236.99 |
| Deaths | North Africa | Sudan | 0 | 1882 | 738 | 155.01 |
| Deaths | North Africa | Sudan | -1 | 1420 | 738 | 92.41 |
| Deaths | North Africa | Sudan | -2 | 1069 | 738 | 44.85 |
| Deaths | North Africa | Sudan | -3 | 802 | 738 | 8.67 |
| Deaths | North Africa | Tunisia | 3 | 839 | 210 | 299.52 |
| Deaths | North Africa | Tunisia | 2 | 638 | 210 | 203.81 |
| Deaths | North Africa | Tunisia | 1 | 484 | 210 | 130.48 |
| Deaths | North Africa | Tunisia | 0 | 367 | 210 | 74.76 |
| Deaths | North Africa | Tunisia | -1 | 277 | 210 | 31.9 |
| Deaths | North Africa | Tunisia | -2 | 208 | 210 | -0.95 |
| Deaths | North Africa | Tunisia | -3 | 156 | 210 | -25.71 |
| Deaths | Southern Africa | Botswana | 3 | 1219 | 253 | 381.82 |
| Deaths | Southern Africa | Botswana | 2 | 927 | 253 | 266.4 |
| Deaths | Southern Africa | Botswana | 1 | 704 | 253 | 178.26 |
| Deaths | Southern Africa | Botswana | 0 | 533 | 253 | 110.67 |
| Deaths | Southern Africa | Botswana | -1 | 402 | 253 | 58.89 |
| Deaths | Southern Africa | Botswana | -2 | 303 | 253 | 19.76 |
| Deaths | Southern Africa | Botswana | -3 | 227 | 253 | -10.28 |
| Deaths | Southern Africa | Eswatini | 3 | 1377 | 269 | 411.9 |
| Deaths | Southern Africa | Eswatini | 2 | 1048 | 269 | 289.59 |
| Deaths | Southern Africa | Eswatini | 1 | 795 | 269 | 195.54 |
| Deaths | Southern Africa | Eswatini | 0 | 602 | 269 | 123.79 |
| Deaths | Southern Africa | Eswatini | -1 | 454 | 269 | 68.77 |
| Deaths | Southern Africa | Eswatini | -2 | 342 | 269 | 27.14 |
| Deaths | Southern Africa | Eswatini | -3 | 256 | 269 | -4.83 |
| Deaths | Southern Africa | Lesotho | 3 | 1494 | 413 | 261.74 |
| Deaths | Southern Africa | Lesotho | 2 | 1137 | 413 | 175.3 |
| Deaths | Southern Africa | Lesotho | 1 | 863 | 413 | 108.96 |
| Deaths | Southern Africa | Lesotho | 0 | 653 | 413 | 58.11 |
| Deaths | Southern Africa | Lesotho | -1 | 493 | 413 | 19.37 |
| Deaths | Southern Africa | Lesotho | -2 | 371 | 413 | -10.17 |
| Deaths | Southern Africa | Lesotho | -3 | 278 | 413 | -32.69 |
| Deaths | Southern Africa | Malawi | 3 | 21367 | 3340 | 539.73 |
| Deaths | Southern Africa | Malawi | 2 | 16259 | 3340 | 386.8 |
| Deaths | Southern Africa | Malawi | 1 | 12339 | 3340 | 269.43 |
| Deaths | Southern Africa | Malawi | 0 | 9339 | 3340 | 179.61 |
| Deaths | Southern Africa | Malawi | -1 | 7048 | 3340 | 111.02 |
| Deaths | Southern Africa | Malawi | -2 | 5304 | 3340 | 58.8 |
| Deaths | Southern Africa | Malawi | -3 | 3980 | 3340 | 19.16 |
| Deaths | Southern Africa | Namibia | 3 | 995 | 203 | 390.15 |
| Deaths | Southern Africa | Namibia | 2 | 757 | 203 | 272.91 |
| Deaths | Southern Africa | Namibia | 1 | 574 | 203 | 182.76 |
| Deaths | Southern Africa | Namibia | 0 | 435 | 203 | 114.29 |
| Deaths | Southern Africa | Namibia | -1 | 328 | 203 | 61.58 |
| Deaths | Southern Africa | Namibia | -2 | 247 | 203 | 21.67 |
| Deaths | Southern Africa | Namibia | -3 | 185 | 203 | -8.87 |
| Deaths | Southern Africa | South Africa | 3 | 23994 | 5976 | 301.51 |
| Deaths | Southern Africa | South Africa | 2 | 18258 | 5976 | 205.52 |
| Deaths | Southern Africa | South Africa | 1 | 13856 | 5976 | 131.86 |
| Deaths | Southern Africa | South Africa | 0 | 10487 | 5976 | 75.49 |
| Deaths | Southern Africa | South Africa | -1 | 7915 | 5976 | 32.45 |
| Deaths | Southern Africa | South Africa | -2 | 5956 | 5976 | -0.33 |
| Deaths | Southern Africa | South Africa | -3 | 4470 | 5976 | -25.2 |
| Deaths | Southern Africa | Zimbabwe | 3 | 13798 | 2318 | 495.25 |
| Deaths | Southern Africa | Zimbabwe | 2 | 10500 | 2318 | 352.98 |
| Deaths | Southern Africa | Zimbabwe | 1 | 7969 | 2318 | 243.79 |
| Deaths | Southern Africa | Zimbabwe | 0 | 6031 | 2318 | 160.18 |
| Deaths | Southern Africa | Zimbabwe | -1 | 4552 | 2318 | 96.38 |
| Deaths | Southern Africa | Zimbabwe | -2 | 3425 | 2318 | 47.76 |
| Deaths | Southern Africa | Zimbabwe | -3 | 2570 | 2318 | 10.87 |
| Deaths | East Africa | Burundi | 3 | 7520 | 1081 | 595.65 |
| Deaths | East Africa | Burundi | 2 | 5722 | 1081 | 429.32 |
| Deaths | East Africa | Burundi | 1 | 4343 | 1081 | 301.76 |
| Deaths | East Africa | Burundi | 0 | 3287 | 1081 | 204.07 |
| Deaths | East Africa | Burundi | -1 | 2481 | 1081 | 129.51 |
| Deaths | East Africa | Burundi | -2 | 1867 | 1081 | 72.71 |
| Deaths | East Africa | Burundi | -3 | 1401 | 1081 | 29.6 |
| Deaths | East Africa | Comoros | 3 | 538 | 102 | 427.45 |
| Deaths | East Africa | Comoros | 2 | 410 | 102 | 301.96 |
| Deaths | East Africa | Comoros | 1 | 311 | 102 | 204.9 |
| Deaths | East Africa | Comoros | 0 | 235 | 102 | 130.39 |
| Deaths | East Africa | Comoros | -1 | 178 | 102 | 74.51 |
| Deaths | East Africa | Comoros | -2 | 134 | 102 | 31.37 |
| Deaths | East Africa | Comoros | -3 | 100 | 102 | -1.96 |
| Deaths | East Africa | Djibouti | 3 | 296 | 54 | 448.15 |
| Deaths | East Africa | Djibouti | 2 | 225 | 54 | 316.67 |
| Deaths | East Africa | Djibouti | 1 | 171 | 54 | 216.67 |
| Deaths | East Africa | Djibouti | 0 | 129 | 54 | 138.89 |
| Deaths | East Africa | Djibouti | -1 | 98 | 54 | 81.48 |
| Deaths | East Africa | Djibouti | -2 | 73 | 54 | 35.19 |
| Deaths | East Africa | Djibouti | -3 | 55 | 54 | 1.85 |
| Deaths | East Africa | Eritrea | 3 | 819 | 150 | 446 |
| Deaths | East Africa | Eritrea | 2 | 624 | 150 | 316 |
| Deaths | East Africa | Eritrea | 1 | 473 | 150 | 215.33 |
| Deaths | East Africa | Eritrea | 0 | 358 | 150 | 138.67 |
| Deaths | East Africa | Eritrea | -1 | 270 | 150 | 80 |
| Deaths | East Africa | Eritrea | -2 | 203 | 150 | 35.33 |
| Deaths | East Africa | Eritrea | -3 | 153 | 150 | 2 |
| Deaths | East Africa | Ethiopia | 3 | 38682 | 5975 | 547.4 |
| Deaths | East Africa | Ethiopia | 2 | 29435 | 5975 | 392.64 |
| Deaths | East Africa | Ethiopia | 1 | 22339 | 5975 | 273.87 |
| Deaths | East Africa | Ethiopia | 0 | 16907 | 5975 | 182.96 |
| Deaths | East Africa | Ethiopia | -1 | 12760 | 5975 | 113.56 |
| Deaths | East Africa | Ethiopia | -2 | 9603 | 5975 | 60.72 |
| Deaths | East Africa | Ethiopia | -3 | 7206 | 5975 | 20.6 |
| Deaths | East Africa | France, La Ré | 3 | 142 | 39 | 264.1 |
| Deaths | East Africa | France, La Ré | 2 | 108 | 39 | 176.92 |
| Deaths | East Africa | France, La Ré | 1 | 82 | 39 | 110.26 |
| Deaths | East Africa | France, La Ré | 0 | 62 | 39 | 58.97 |
| Deaths | East Africa | France, La Ré | -1 | 47 | 39 | 20.51 |
| Deaths | East Africa | France, La Ré | -2 | 35 | 39 | -10.26 |
| Deaths | East Africa | France, La Ré | -3 | 26 | 39 | -33.33 |
| Deaths | East Africa | Kenya | 3 | 20781 | 3591 | 478.7 |
| Deaths | East Africa | Kenya | 2 | 15814 | 3591 | 340.38 |
| Deaths | East Africa | Kenya | 1 | 12001 | 3591 | 234.2 |
| Deaths | East Africa | Kenya | 0 | 9083 | 3591 | 152.94 |
| Deaths | East Africa | Kenya | -1 | 6855 | 3591 | 90.89 |
| Deaths | East Africa | Kenya | -2 | 5159 | 3591 | 43.66 |
| Deaths | East Africa | Kenya | -3 | 3871 | 3591 | 7.8 |
| Deaths | East Africa | Madagascar | 3 | 16859 | 2690 | 526.73 |
| Deaths | East Africa | Madagascar | 2 | 12829 | 2690 | 376.91 |
| Deaths | East Africa | Madagascar | 1 | 9736 | 2690 | 261.93 |
| Deaths | East Africa | Madagascar | 0 | 7369 | 2690 | 173.94 |
| Deaths | East Africa | Madagascar | -1 | 5561 | 2690 | 106.73 |
| Deaths | East Africa | Madagascar | -2 | 4185 | 2690 | 55.58 |
| Deaths | East Africa | Madagascar | -3 | 3141 | 2690 | 16.77 |
| Deaths | East Africa | Mauritius | 3 | 243 | 67 | 262.69 |
| Deaths | East Africa | Mauritius | 2 | 185 | 67 | 176.12 |
| Deaths | East Africa | Mauritius | 1 | 140 | 67 | 108.96 |
| Deaths | East Africa | Mauritius | 0 | 106 | 67 | 58.21 |
| Deaths | East Africa | Mauritius | -1 | 80 | 67 | 19.4 |
| Deaths | East Africa | Mauritius | -2 | 60 | 67 | -10.45 |
| Deaths | East Africa | Mauritius | -3 | 45 | 67 | -32.84 |
| Deaths | East Africa | Mozambique | 3 | 23058 | 4000 | 476.45 |
| Deaths | East Africa | Mozambique | 2 | 17546 | 4000 | 338.65 |
| Deaths | East Africa | Mozambique | 1 | 13316 | 4000 | 232.9 |
| Deaths | East Africa | Mozambique | 0 | 10078 | 4000 | 151.95 |
| Deaths | East Africa | Mozambique | -1 | 7606 | 4000 | 90.15 |
| Deaths | East Africa | Mozambique | -2 | 5724 | 4000 | 43.1 |
| Deaths | East Africa | Mozambique | -3 | 4295 | 4000 | 7.38 |
| Deaths | East Africa | Rwanda | 3 | 3607 | 609 | 492.28 |
| Deaths | East Africa | Rwanda | 2 | 2745 | 609 | 350.74 |
| Deaths | East Africa | Rwanda | 1 | 2083 | 609 | 242.04 |
| Deaths | East Africa | Rwanda | 0 | 1577 | 609 | 158.95 |
| Deaths | East Africa | Rwanda | -1 | 1190 | 609 | 95.4 |
| Deaths | East Africa | Rwanda | -2 | 896 | 609 | 47.13 |
| Deaths | East Africa | Rwanda | -3 | 672 | 609 | 10.34 |
| Deaths | East Africa | Somalia | 3 | 5700 | 919 | 520.24 |
| Deaths | East Africa | Somalia | 2 | 4338 | 919 | 372.03 |
| Deaths | East Africa | Somalia | 1 | 3292 | 919 | 258.22 |
| Deaths | East Africa | Somalia | 0 | 2491 | 919 | 171.06 |
| Deaths | East Africa | Somalia | -1 | 1880 | 919 | 104.57 |
| Deaths | East Africa | Somalia | -2 | 1415 | 919 | 53.97 |
| Deaths | East Africa | Somalia | -3 | 1062 | 919 | 15.56 |
| Deaths | East Africa | Tanzania | 3 | 49594 | 6832 | 625.91 |
| Deaths | East Africa | Tanzania | 2 | 37739 | 6832 | 452.39 |
| Deaths | East Africa | Tanzania | 1 | 28641 | 6832 | 319.22 |
| Deaths | East Africa | Tanzania | 0 | 21677 | 6832 | 217.29 |
| Deaths | East Africa | Tanzania | -1 | 16360 | 6832 | 139.46 |
| Deaths | East Africa | Tanzania | -2 | 12312 | 6832 | 80.21 |
| Deaths | East Africa | Tanzania | -3 | 9238 | 6832 | 35.22 |
| Deaths | East Africa | Uganda | 3 | 31947 | 4782 | 568.07 |
| Deaths | East Africa | Uganda | 2 | 24310 | 4782 | 408.36 |
| Deaths | East Africa | Uganda | 1 | 18449 | 4782 | 285.8 |
| Deaths | East Africa | Uganda | 0 | 13963 | 4782 | 191.99 |
| Deaths | East Africa | Uganda | -1 | 10538 | 4782 | 120.37 |
| Deaths | East Africa | Uganda | -2 | 7931 | 4782 | 65.85 |
| Deaths | East Africa | Uganda | -3 | 5951 | 4782 | 24.45 |
| Deaths | East Africa | Zambia | 3 | 16862 | 2285 | 637.94 |
| Deaths | East Africa | Zambia | 2 | 12831 | 2285 | 461.53 |
| Deaths | East Africa | Zambia | 1 | 9738 | 2285 | 326.17 |
| Deaths | East Africa | Zambia | 0 | 7370 | 2285 | 222.54 |
| Deaths | East Africa | Zambia | -1 | 5562 | 2285 | 143.41 |
| Deaths | East Africa | Zambia | -2 | 4186 | 2285 | 83.19 |
| Deaths | East Africa | Zambia | -3 | 3141 | 2285 | 37.46 |
| Deaths | West Africa | Angola | 3 | 12015 | 1715 | 600.58 |
| Deaths | West Africa | Angola | 2 | 9143 | 1715 | 433.12 |
| Deaths | West Africa | Angola | 1 | 6939 | 1715 | 304.61 |
| Deaths | West Africa | Angola | 0 | 5252 | 1715 | 206.24 |
| Deaths | West Africa | Angola | -1 | 3963 | 1715 | 131.08 |
| Deaths | West Africa | Angola | -2 | 2983 | 1715 | 73.94 |
| Deaths | West Africa | Angola | -3 | 2238 | 1715 | 30.5 |
| Deaths | West Africa | Benin | 3 | 2753 | 475 | 479.58 |
| Deaths | West Africa | Benin | 2 | 2095 | 475 | 341.05 |
| Deaths | West Africa | Benin | 1 | 1590 | 475 | 234.74 |
| Deaths | West Africa | Benin | 0 | 1203 | 475 | 153.26 |
| Deaths | West Africa | Benin | -1 | 908 | 475 | 91.16 |
| Deaths | West Africa | Benin | -2 | 683 | 475 | 43.79 |
| Deaths | West Africa | Benin | -3 | 513 | 475 | 8 |
| Deaths | West Africa | Burkina Faso | 3 | 4849 | 775 | 525.68 |
| Deaths | West Africa | Burkina Faso | 2 | 3690 | 775 | 376.13 |
| Deaths | West Africa | Burkina Faso | 1 | 2801 | 775 | 261.42 |
| Deaths | West Africa | Burkina Faso | 0 | 2120 | 775 | 173.55 |
| Deaths | West Africa | Burkina Faso | -1 | 1600 | 775 | 106.45 |
| Deaths | West Africa | Burkina Faso | -2 | 1204 | 775 | 55.35 |
| Deaths | West Africa | Burkina Faso | -3 | 903 | 775 | 16.52 |
| Deaths | West Africa | Cameroon | 3 | 11489 | 1837 | 525.42 |
| Deaths | West Africa | Cameroon | 2 | 8742 | 1837 | 375.88 |
| Deaths | West Africa | Cameroon | 1 | 6635 | 1837 | 261.19 |
| Deaths | West Africa | Cameroon | 0 | 5021 | 1837 | 173.33 |
| Deaths | West Africa | Cameroon | -1 | 3790 | 1837 | 106.31 |
| Deaths | West Africa | Cameroon | -2 | 2852 | 1837 | 55.25 |
| Deaths | West Africa | Cameroon | -3 | 2140 | 1837 | 16.49 |
| Deaths | West Africa | Cape Verde | 3 | 136 | 27 | 403.7 |
| Deaths | West Africa | Cape Verde | 2 | 103 | 27 | 281.48 |
| Deaths | West Africa | Cape Verde | 1 | 78 | 27 | 188.89 |
| Deaths | West Africa | Cape Verde | 0 | 59 | 27 | 118.52 |
| Deaths | West Africa | Cape Verde | -1 | 45 | 27 | 66.67 |
| Deaths | West Africa | Cape Verde | -2 | 34 | 27 | 25.93 |
| Deaths | West Africa | Cape Verde | -3 | 25 | 27 | -7.41 |
| Deaths | West Africa | Côte d'Ivoire | 3 | 9106 | 1461 | 523.27 |
| Deaths | West Africa | Côte d'Ivoire | 2 | 6929 | 1461 | 374.26 |
| Deaths | West Africa | Côte d'Ivoire | 1 | 5258 | 1461 | 259.89 |
| Deaths | West Africa | Côte d'Ivoire | 0 | 3980 | 1461 | 172.42 |
| Deaths | West Africa | Côte d'Ivoire | -1 | 3004 | 1461 | 105.61 |
| Deaths | West Africa | Côte d'Ivoire | -2 | 2260 | 1461 | 54.69 |
| Deaths | West Africa | Côte d'Ivoire | -3 | 1696 | 1461 | 16.08 |
| Deaths | West Africa | Gambia | 3 | 1325 | 204 | 549.51 |
| Deaths | West Africa | Gambia | 2 | 1008 | 204 | 394.12 |
| Deaths | West Africa | Gambia | 1 | 765 | 204 | 275 |
| Deaths | West Africa | Gambia | 0 | 579 | 204 | 183.82 |
| Deaths | West Africa | Gambia | -1 | 437 | 204 | 114.22 |
| Deaths | West Africa | Gambia | -2 | 329 | 204 | 61.27 |
| Deaths | West Africa | Gambia | -3 | 247 | 204 | 21.08 |
| Deaths | West Africa | Ghana | 3 | 10529 | 1815 | 480.11 |
| Deaths | West Africa | Ghana | 2 | 8012 | 1815 | 341.43 |
| Deaths | West Africa | Ghana | 1 | 6081 | 1815 | 235.04 |
| Deaths | West Africa | Ghana | 0 | 4602 | 1815 | 153.55 |
| Deaths | West Africa | Ghana | -1 | 3473 | 1815 | 91.35 |
| Deaths | West Africa | Ghana | -2 | 2614 | 1815 | 44.02 |
| Deaths | West Africa | Ghana | -3 | 1961 | 1815 | 8.04 |
| Deaths | West Africa | Guinea | 3 | 9284 | 1695 | 447.73 |
| Deaths | West Africa | Guinea | 2 | 7065 | 1695 | 316.81 |
| Deaths | West Africa | Guinea | 1 | 5362 | 1695 | 216.34 |
| Deaths | West Africa | Guinea | 0 | 4058 | 1695 | 139.41 |
| Deaths | West Africa | Guinea | -1 | 3063 | 1695 | 80.71 |
| Deaths | West Africa | Guinea | -2 | 2305 | 1695 | 35.99 |
| Deaths | West Africa | Guinea | -3 | 1729 | 1695 | 2.01 |
| Deaths | West Africa | Guinea-Bissau | 3 | 934 | 157 | 494.9 |
| Deaths | West Africa | Guinea-Bissau | 2 | 711 | 157 | 352.87 |
| Deaths | West Africa | Guinea-Bissau | 1 | 539 | 157 | 243.31 |
| Deaths | West Africa | Guinea-Bissau | 0 | 408 | 157 | 159.87 |
| Deaths | West Africa | Guinea-Bissau | -1 | 308 | 157 | 96.18 |
| Deaths | West Africa | Guinea-Bissau | -2 | 232 | 157 | 47.77 |
| Deaths | West Africa | Guinea-Bissau | -3 | 174 | 157 | 10.83 |
| Deaths | West Africa | Liberia | 3 | 2474 | 478 | 417.57 |
| Deaths | West Africa | Liberia | 2 | 1882 | 478 | 293.72 |
| Deaths | West Africa | Liberia | 1 | 1429 | 478 | 198.95 |
| Deaths | West Africa | Liberia | 0 | 1081 | 478 | 126.15 |
| Deaths | West Africa | Liberia | -1 | 816 | 478 | 70.71 |
| Deaths | West Africa | Liberia | -2 | 614 | 478 | 28.45 |
| Deaths | West Africa | Liberia | -3 | 461 | 478 | -3.56 |
| Deaths | West Africa | Mali | 3 | 9041 | 1431 | 531.8 |
| Deaths | West Africa | Mali | 2 | 6880 | 1431 | 380.78 |
| Deaths | West Africa | Mali | 1 | 5221 | 1431 | 264.85 |
| Deaths | West Africa | Mali | 0 | 3952 | 1431 | 176.17 |
| Deaths | West Africa | Mali | -1 | 2982 | 1431 | 108.39 |
| Deaths | West Africa | Mali | -2 | 2244 | 1431 | 56.81 |
| Deaths | West Africa | Mali | -3 | 1684 | 1431 | 17.68 |
| Deaths | West Africa | Mauritania | 3 | 1730 | 302 | 472.85 |
| Deaths | West Africa | Mauritania | 2 | 1316 | 302 | 335.76 |
| Deaths | West Africa | Mauritania | 1 | 999 | 302 | 230.79 |
| Deaths | West Africa | Mauritania | 0 | 756 | 302 | 150.33 |
| Deaths | West Africa | Mauritania | -1 | 571 | 302 | 89.07 |
| Deaths | West Africa | Mauritania | -2 | 429 | 302 | 42.05 |
| Deaths | West Africa | Mauritania | -3 | 322 | 302 | 6.62 |
| Deaths | West Africa | Niger | 3 | 2990 | 440 | 579.55 |
| Deaths | West Africa | Niger | 2 | 2275 | 440 | 417.05 |
| Deaths | West Africa | Niger | 1 | 1726 | 440 | 292.27 |
| Deaths | West Africa | Niger | 0 | 1307 | 440 | 197.05 |
| Deaths | West Africa | Niger | -1 | 986 | 440 | 124.09 |
| Deaths | West Africa | Niger | -2 | 742 | 440 | 68.64 |
| Deaths | West Africa | Niger | -3 | 557 | 440 | 26.59 |
| Deaths | West Africa | Nigeria | 3 | 40190 | 7093 | 466.61 |
| Deaths | West Africa | Nigeria | 2 | 30583 | 7093 | 331.17 |
| Deaths | West Africa | Nigeria | 1 | 23210 | 7093 | 227.22 |
| Deaths | West Africa | Nigeria | 0 | 17566 | 7093 | 147.65 |
| Deaths | West Africa | Nigeria | -1 | 13257 | 7093 | 86.9 |
| Deaths | West Africa | Nigeria | -2 | 9977 | 7093 | 40.66 |
| Deaths | West Africa | Nigeria | -3 | 7487 | 7093 | 5.55 |
| Deaths | West Africa | Senegal | 3 | 8162 | 1327 | 515.07 |
| Deaths | West Africa | Senegal | 2 | 6211 | 1327 | 368.05 |
| Deaths | West Africa | Senegal | 1 | 4713 | 1327 | 255.16 |
| Deaths | West Africa | Senegal | 0 | 3567 | 1327 | 168.8 |
| Deaths | West Africa | Senegal | -1 | 2692 | 1327 | 102.86 |
| Deaths | West Africa | Senegal | -2 | 2026 | 1327 | 52.68 |
| Deaths | West Africa | Senegal | -3 | 1520 | 1327 | 14.54 |
| Deaths | West Africa | Sierra Leone | 3 | 1682 | 292 | 476.03 |
| Deaths | West Africa | Sierra Leone | 2 | 1280 | 292 | 338.36 |
| Deaths | West Africa | Sierra Leone | 1 | 972 | 292 | 232.88 |
| Deaths | West Africa | Sierra Leone | 0 | 735 | 292 | 151.71 |
| Deaths | West Africa | Sierra Leone | -1 | 555 | 292 | 90.07 |
| Deaths | West Africa | Sierra Leone | -2 | 418 | 292 | 43.15 |
| Deaths | West Africa | Sierra Leone | -3 | 313 | 292 | 7.19 |
| Deaths | West Africa | Togo | 3 | 1917 | 334 | 473.95 |
| Deaths | West Africa | Togo | 2 | 1459 | 334 | 336.83 |
| Deaths | West Africa | Togo | 1 | 1107 | 334 | 231.44 |
| Deaths | West Africa | Togo | 0 | 838 | 334 | 150.9 |
| Deaths | West Africa | Togo | -1 | 632 | 334 | 89.22 |
| Deaths | West Africa | Togo | -2 | 476 | 334 | 42.51 |
| Deaths | West Africa | Togo | -3 | 357 | 334 | 6.89 |
| Deaths | Central Africa | Central Africa Rep | 3 | 1231 | 240 | 412.92 |
| Deaths | Central Africa | Central Africa Rep | 2 | 937 | 240 | 290.42 |
| Deaths | Central Africa | Central Africa Rep | 1 | 711 | 240 | 196.25 |
| Deaths | Central Africa | Central Africa Rep | 0 | 538 | 240 | 124.17 |
| Deaths | Central Africa | Central Africa Rep | -1 | 406 | 240 | 69.17 |
| Deaths | Central Africa | Central Africa Rep | -2 | 306 | 240 | 27.5 |
| Deaths | Central Africa | Central Africa Rep | -3 | 229 | 240 | -4.58 |
| Deaths | Central Africa | Chad | 3 | 5268 | 841 | 526.4 |
| Deaths | Central Africa | Chad | 2 | 4009 | 841 | 376.69 |
| Deaths | Central Africa | Chad | 1 | 3042 | 841 | 261.71 |
| Deaths | Central Africa | Chad | 0 | 2302 | 841 | 173.72 |
| Deaths | Central Africa | Chad | -1 | 1738 | 841 | 106.66 |
| Deaths | Central Africa | Chad | -2 | 1308 | 841 | 55.53 |
| Deaths | Central Africa | Chad | -3 | 981 | 841 | 16.65 |
| Deaths | Central Africa | Congo, Republic of | 3 | 1381 | 248 | 456.85 |
| Deaths | Central Africa | Congo, Republic of | 2 | 1051 | 248 | 323.79 |
| Deaths | Central Africa | Congo, Republic of | 1 | 797 | 248 | 221.37 |
| Deaths | Central Africa | Congo, Republic of | 0 | 604 | 248 | 143.55 |
| Deaths | Central Africa | Congo, Republic of | -1 | 456 | 248 | 83.87 |
| Deaths | Central Africa | Congo, Republic of | -2 | 343 | 248 | 38.31 |
| Deaths | Central Africa | Congo, Republic of | -3 | 257 | 248 | 3.63 |
| Deaths | Central Africa | DR Congo | 3 | 37342 | 6187 | 503.56 |
| Deaths | Central Africa | DR Congo | 2 | 28416 | 6187 | 359.29 |
| Deaths | Central Africa | DR Congo | 1 | 21565 | 6187 | 248.55 |
| Deaths | Central Africa | DR Congo | 0 | 16321 | 6187 | 163.8 |
| Deaths | Central Africa | DR Congo | -1 | 12318 | 6187 | 99.09 |
| Deaths | Central Africa | DR Congo | -2 | 9270 | 6187 | 49.83 |
| Deaths | Central Africa | DR Congo | -3 | 6956 | 6187 | 12.43 |
| Deaths | Central Africa | Equatorial Guinea | 3 | 623 | 76 | 719.74 |
| Deaths | Central Africa | Equatorial Guinea | 2 | 474 | 76 | 523.68 |
| Deaths | Central Africa | Equatorial Guinea | 1 | 360 | 76 | 373.68 |
| Deaths | Central Africa | Equatorial Guinea | 0 | 272 | 76 | 257.89 |
| Deaths | Central Africa | Equatorial Guinea | -1 | 206 | 76 | 171.05 |
| Deaths | Central Africa | Equatorial Guinea | -2 | 155 | 76 | 103.95 |
| Deaths | Central Africa | Equatorial Guinea | -3 | 116 | 76 | 52.63 |
| Deaths | Central Africa | Gabon | 3 | 805 | 139 | 479.14 |
| Deaths | Central Africa | Gabon | 2 | 613 | 139 | 341.01 |
| Deaths | Central Africa | Gabon | 1 | 465 | 139 | 234.53 |
| Deaths | Central Africa | Gabon | 0 | 352 | 139 | 153.24 |
| Deaths | Central Africa | Gabon | -1 | 266 | 139 | 91.37 |
| Deaths | Central Africa | Gabon | -2 | 200 | 139 | 43.88 |
| Deaths | Central Africa | Gabon | -3 | 150 | 139 | 7.91 |
| Deaths | Central Africa | Sao Tome | 3 | 57 | 10 | 470 |
| Deaths | Central Africa | Sao Tome | 2 | 43 | 10 | 330 |
| Deaths | Central Africa | Sao Tome | 1 | 33 | 10 | 230 |
| Deaths | Central Africa | Sao Tome | 0 | 25 | 10 | 150 |
| Deaths | Central Africa | Sao Tome | -1 | 19 | 10 | 90 |
| Deaths | Central Africa | Sao Tome | -2 | 14 | 10 | 40 |
| Deaths | Central Africa | Sao Tome | -3 | 11 | 10 | 10 |

The percentage (%) represents the change in new cases or deaths in 2050. The scale presents the annual change (%) in the new case or death number from the baseline year 2022. The data was obtained from the GLOBOCAN database in 2022.
